# Integrated use of regional weather forecasting and crop modeling for water stress assessment on rice yield

**DOI:** 10.1038/s41598-022-19750-z

**Published:** 2022-10-10

**Authors:** T. Rajasivaranjan, Aavudai Anandhi, N. R. Patel, Masoud Irannezhad, C. V. Srinivas, Kumar Veluswamy, U. Surendran, P. Raja

**Affiliations:** 1 Indian Institute of Remote Sensing, Dehradun, 248001 India; 2 Biological Systems Engineering, Florida Agricultural and Mechanical University, Tallahassee, FL 32307 USA; 3 Water, Energy and Environmental Engineering Research Unit, Faculty of Technology, University of Oulu, 90014 Oulu, Finland; 4 Environmental Assessment Division, Indira Gandhi Centre for Atomic Research, Homi Bhabha National Institute, Kalpakkam, Tamil Nadu India; 5 Department of Agricultural Engineering, TNAU: Agricultural College and Research Institute - Madurai Campus, Madurai, 625 104 India; 6 Water Management (Agriculture) Division, Centre for Water Resources Development and Management, Kunnamangalam, Kozhikode, 673571 Kerala India; 7 ICAR-Indian Institute of Soil and Water Conservation, R.C, Udhagamandalam, 643 004 Tamil Nadu India

**Keywords:** Plant sciences, Climate sciences, Hydrology, Mathematics and computing

## Abstract

This study evaluated the effects of water stress on rice yield over Punjab and Haryana across North India by integrating Weather Research Forecasting (WRF) and Decision Support System for Agrotechnology Transfer (DSSAT) models. Indian Remote Sensing Satellite datasets were used to define land use/land cover in WRF. The accuracy of simulated rainfall and temperature over Punjab and Haryana was evaluated against Tropical Rainfall Measuring Mission and automated weather station data of Indian Space Research Organization, respectively. Data from WRF was used as weather input to DSSAT to simulate rice yield in Punjab and Haryana for 2009 and 2014. After simulated yield has been evaluated against district-level observed yield, the water balance components within the DSSAT model were used to analyze the impact of water stress on rice yield. The correlation (R^2^) between the crop water stress factor and the rice yield anomaly at the vegetative and reproductive stage was 0.64 and 0.52 for Haryana and 0.73 and 0.68 for Punjab, respectively. Severe water stress during the flowering to maturity stage inflicted devastating effects on yield. The study concludes that the regional climate simulations can be potentially used for early water stress prediction and its impact on rice yield.

## Introduction

Indian agriculture is significantly reliant on the southwest summer (Jun–Sep) monsoon performance, and primarily involves small-scale farming approaches that employ rudimentary technologies within a mixed-crop-livestock model. A few regional factors like Eurasian snow cover, soil moisture, land and sea temperatures, and large-scale climate teleconnections (e.g., the El Niño-Southern Oscillation or ENSO influence the Indian Summer Monsoon circulation and associated rainfall pattern^1–4^. A delayed monsoon onset, a prolonged break in rainfall during July and August, early withdrawal of the monsoon, and uneven distribution of rainfall, especially in the low rainfall belts, make India highly vulnerable to droughts. The country has observed drought with varying scales, longevity, and magnitude. During the past 112 years, 14 drought events have occurred, affecting 1.061 billion people and incurring US$2.441 billion in economic loss^5^. For instance, an estimated 19% fall in food grain production was observed after the 1966–1967 drought. The drought of 1972–1973 reduced the food grains production from 108 to 95 million tons and caused a loss of about $400 million. Drought in India in 1987 caused damage to 58.6 million hectares of the cropped area^6^, thereby strongly impacting the food security and sustenance of livelihoods of over 285 million people^7,8^. The food grain production during the 2002 drought had a fall by 38 million tons from 2001 (212 million tons) to 2002 (174 million tons), contributing to a 3.2% reduction in India's agricultural GDP^5,9^. The rainfall deficit for two consecutive years has had its cumulative impact, affecting more than 330 million people in India and the scale of the crisis is unprecedented. More than 250 thousand villages in 266 districts across 11 states have been severely affected, not just in terms of non-availability of water but also in agricultural damage and livelihood loss. Hence the occurrence of drought is a common phenomenon, and it is not an exception anymore under the scenario of climate change and climate variability.

Drought develops gradually over a prolonged period and impacts the full water cycle from the commencement of precipitation deficit, including reservoir levels, lake levels, groundwater, soil moisture, and streamflow. All of this has significant implications for agricultural production, and every aspect needs consideration for effective drought management practices. Agricultural drought initiation may be a slow process. Still, its persistence will have a severe impact on crop production. Hence, a country like India needs to acquire more capacity to monitor and predict all kinds of droughts and climate-related hazards on all scales and times for taking proper adaptation and mitigation measures^10^. There is a requirement to develop an in-depth understanding of agricultural droughts' anatomy to comprehend better moisture supply and demand within cultivation practices^11^.

Crop models are perceived to represent practical tools that can enhance agricultural drought management by providing a simulation of the physiological processes involved in the interactions between the soil, plants, and atmosphere. Crop modeling studies gained importance in recent times^12,13^ as they provide quantitative estimates of climate-crop impacts. The gathered data can be utilized to delineate the yield gap, generate forecasts for future yields, and develop meaningful insights that can contribute to more effective decisions^14^. One of the benefits of crop models is that they offer data that quantify crop performance variability in the climate conditions observed from season to season and make it possible to understand better the long-term implications of climate change and the land use options^15^. In this regard, crop models deliver an objective method of developing statistics that quantify droughts' possible implications for crop development and yield. In general, all the crop simulation models (viz*.,* SUCROS IBSNAT models of DSSAT and the APSIM model) require climate-related information as an input.

Shortage of water/water stress causes soil moisture deficit, which will have an adverse effect on vegetation and result in low agricultural production. Also, it has a direct impact on animals and humans^10^. Meta-analysis on the impact of droughts on rice (55 papers) and wheat (60 papers) showed that drought decreased the agronomic traits differently between rice and wheat among varying growth stages^16^. Even though several studies have been conducted to quantify the effects of water stress, the evaluation and monitoring and its impact on crop production have been limited. Few studies highlighted the need to develop drought monitoring and prediction systems (DMAPS) at regional and global scales^17^.

Dynamical downscaling involves the application of high-resolution regional models with outputs from global climate models to make climate predictions at a local scale for planning purposes. Regional climate models have been successfully used for the simulation of the monsoon regional rainfall patterns as they better represent the effects of regional topography and physical processes such as land-surface energy fluxes, vertical flux transport in the boundary layer, convection, and cloud microphysical processes compared to the coarse resolution global models^18–23^. The regional climate models (RCM) using the downscaling approach have accurately projected climate on a regional scale^18,24^ for climate impact assessment and societal use. Recent studies using WRF^25,26^ showed that the WRF model could produce reasonable simulations of Indian summer monsoon precipitation climatology in different climate zones of India and the rainfall characteristics during the onset phase.

Recent developments in numerical weather prediction (NWP) have significantly improved monsoon forecasting. Prediction of intra-seasonal variations is crucial for effective agricultural planning. Improvement in NWP techniques and the ability to estimate climate at various lead times varying from days to whole seasons has prompted significant interest in the advanced assessment of the impacts of water stress and the relative reduction in yield to enhance drought management and strategies at the policy level. Several studies have used the WRF model to examine energy exchange processes between land and the atmosphere over different regions^27–32^. These studies highlight that WRF shows a reasonable performance over the various region. Hence, the overall aim of this study was to integrate regional weather forecasting and crop models for quantifying the impacts of water shortage on food grain production.

The specific objectives were to (1) understand better the extent to which the WRF model can effectively simulate the Indian Summer Monsoon regional climatic features with ingestion of Indian National Satellite (INSAT) derived land use-land cover data and (2) couple the WRF and the Decision Support System Agrotechnology Transfer (DSSAT) crop simulation models to study the spatiotemporal repercussions of crop-specific water stress and the resulting loss in rice yield for 2009 and 2014 in Punjab and Haryana states in India. The findings of this study lay a foundation for predicting future impacts of climate and land use-land cover changes on local, regional, and global food security, particularly during agricultural drought periods, as well as acting towards achieving the 2030 United Nations Agenda of Sustainable Development.

## Materials and methods

### Study area description

**Figure 1:**
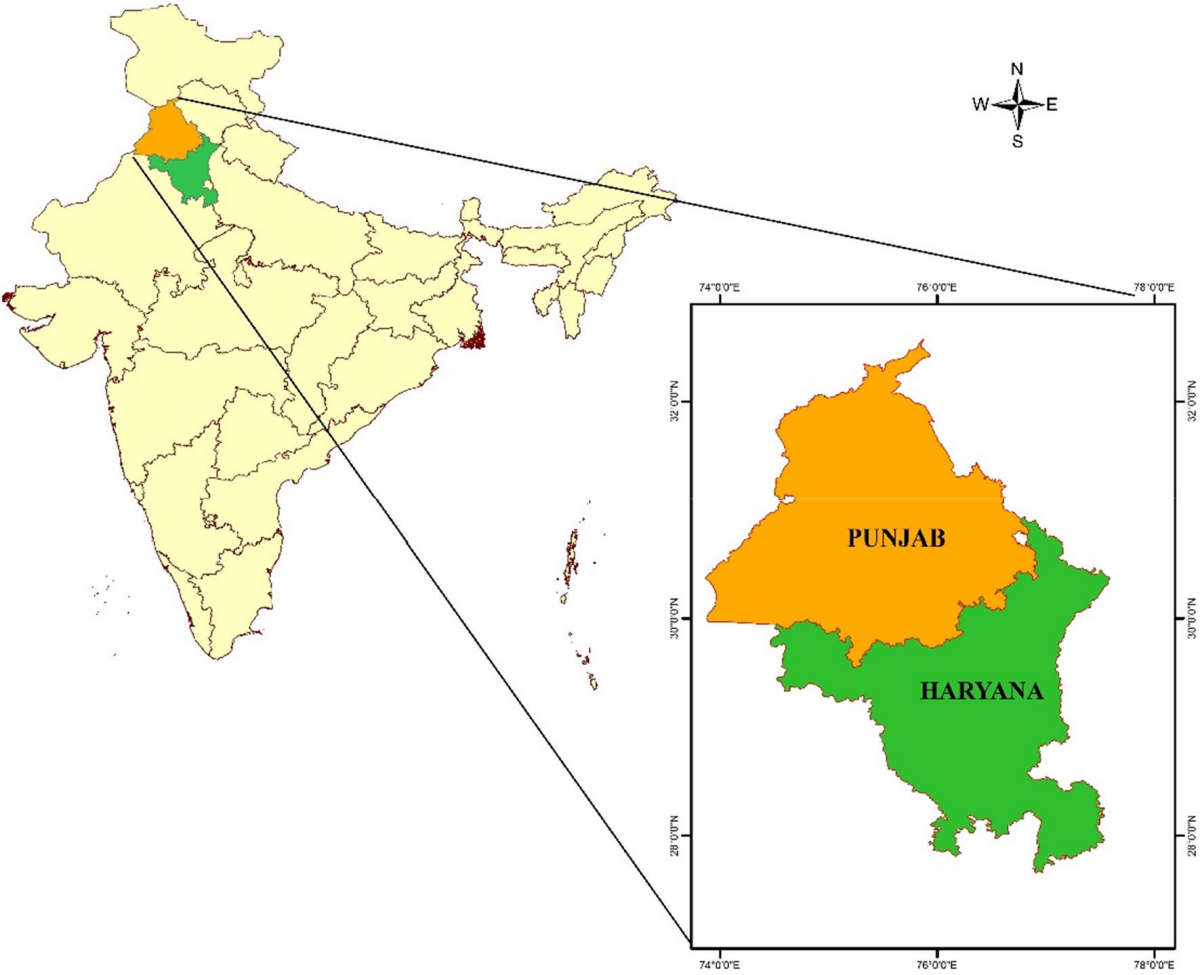
The geographical locations of both Punjab and Haryana states (India) selected for this study (Map created at IIRS—https://www.iirs.gov.in/inhouselaboratories).

Haryana and Punjab states in northern India were selected for the current study as they comprise the food bowl of India (Fig. 1). The state of Haryana extending from 27°39′ N, 74°27′ E to 30°55′ N, 77°36′ E covers a total geographical area of 4.421 million hectares, forming about 1.35 percent of the total geographical area (TGA) of the country. The state of Punjab extends from 29°30′ N, 73°55′ E to 32°32′ N, 76°50′ E. It covers a total geographical area of 5.036 million hectares, forming about 1.54 percent of India’s TGA.

### WRF and simulation data description

In the current study, dynamical downscaling experiments are performed by resolving mesoscale forcings associated with coastlines, mountains, lakes and land use/vegetation characteristics which play a vital role in local climate and the precipitation distributions using high-resolution WRF model^33,34^. The current study employed the Advanced Research Weather Research Forecasting (WRF-ARW) version 3.6 for regional climate simulations. WRF is a mesoscale model, with non-hydrostatic dynamics and terrain-following vertical coordinate designed as a next-generation model used for both operational forecasting and atmospheric research^35^. WRF has been used for regional climate studies over different parts of India^25,26,36,37^. The simulations of WRF for two years, 2009 and 2014, were used to examine the correlation between water stress and crop yield loss. The year 2009 was chosen since that was a drought-affected year in most parts of India, whereas the rainfall during 2014 was slightly below average.

**Figure 2:**
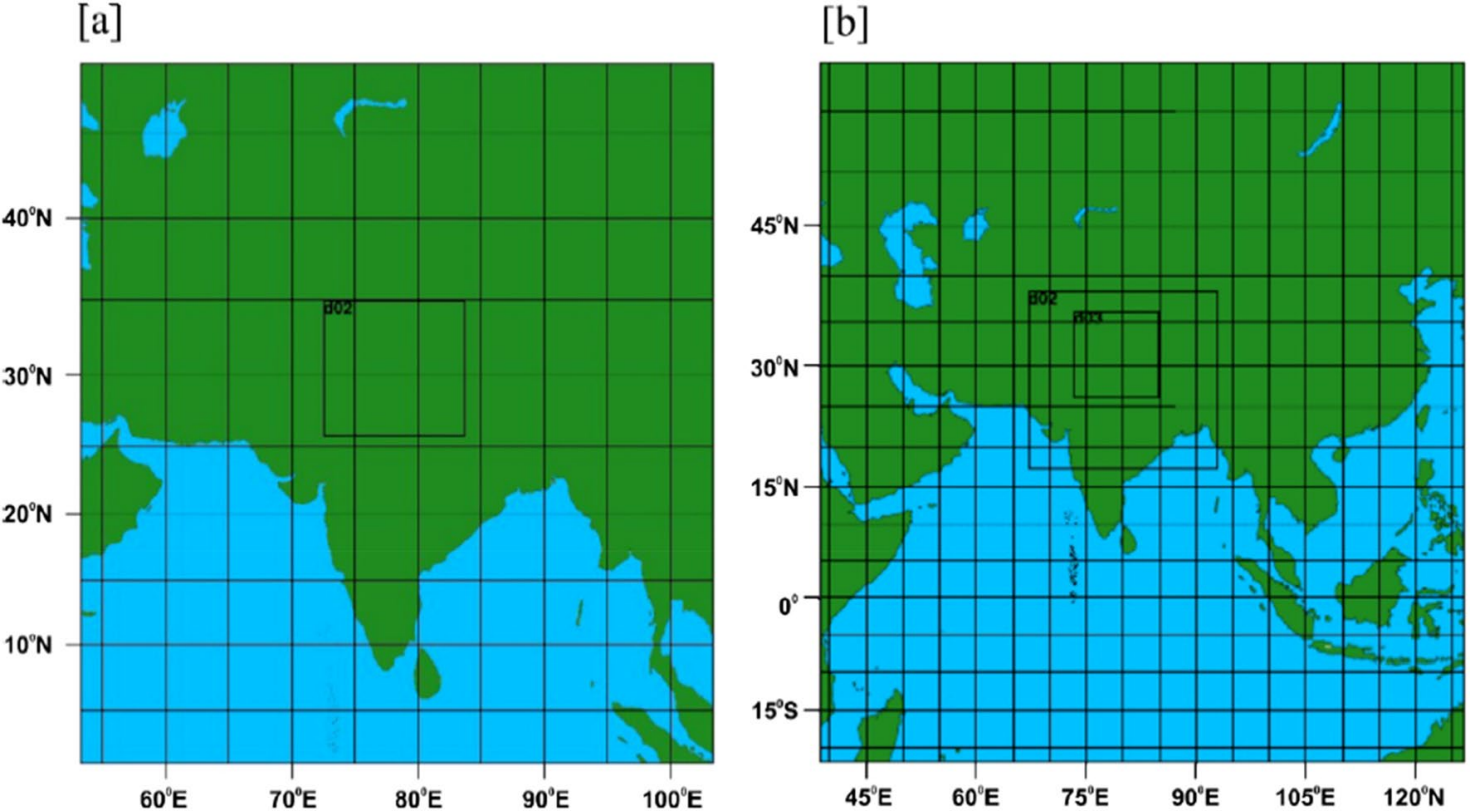
WRF domain setup. (**a**) 2009—two nested domains with horizontal resolution of 30 and 10 km; (**b**) 2014—three nested domains with horizontal resolution of 90 km, 30 and 10 km (Map created at IIRS—https://www.iirs.gov.in/inhouselaboratories).

The model for 2009 simulation was configured with two nested domains (187 × 180; 99 × 99 grids) with grid spacings of 30 km and 10 km and 37 vertical levels and with three domains (99 × 76; 95 × 110; 121 × 110 grids) with grid spacings of 90 km, 30 km, and 10 km and 27 vertical levels for 2014 (Fig. 2). This model set-up was adopted differently for 2009 and 2014 to suitably downscale from Climate Forecast System Reanalysis (CFSR) for 2009 and the meteorological analysis data of Final Analysis (FNL) for 2014.

The six-hourly 1° × 1° National Centres for Environmental Prediction (NCEP) FNL data^38^ was employed to derive the initial and boundary conditions for the simulation^39^. The model initialization fields include the 3D data of U and V components of wind, temperature, relative humidity, geopotential height, 2D data of surface pressure, mean sea-level pressure, 2 m temperature, 2 m relative humidity, 10 m U and V components of wind, soil moisture and soil temperature in different depths, soil height, water equivalent snow depth, and sea ice. The lateral boundary conditions (LBC) were updated every six hours. The Sea Surface Temperature (SST) is an important lower boundary condition for air-sea flux calculation in regional climate simulations. In this study the SST boundary conditions to WRF model for simulations of both 2009 and 2014 are updated from the daily NOAA/NCEP real-time global (RTG) high-resolution (1/12 degree) analysis SST based on the variation interpolation of the most recent 24-h buoy and ship, NOAA AVHRR satellite observed SST data sets^40^. The model is integrated with the SST input from daily RTG SST analyses, interpolated every 6 h using WRF pre-processing system (WPS), from initialization to completion. The topography is defined from the USGS terrain height arc 30 s data, and the soil types are defined from the Food and Agriculture Organization (FAO) 17 category arc 30 s data. The data sets used to define the land use/land cover are given in “Land use land cover (LULC) information” section. The model is initialized at time zone of 0000 Coordinated Universal Time (UTC) on 1 May and integrated until 0000 UTC on 30 November for each year. The first 30- days of simulation in both cases was considered as spin-up period for model adjustment to regional topography and hence was excluded from the analysis.

#### Physical parameterization

The physical parameterization schemes used for the model simulations consisted of WRF Single-Moment 5-class (WSM5) scheme for microphysics, MM5 scheme for the surface layer, Yonsei University non-local PBL scheme for turbulent diffusion, Rapid Radiative Transfer Model (RRTM) scheme for long-wave radiation^41^, and the Dudhia^42^ scheme for short-wave radiation^28,43^. For land surface energy and moisture transport processes, the unified Noah Land Surface Model (LSM) was used^44,45^. The Noah LSM solves the soil thermal diffusivity and hydrology equations with explicit treatment of a plant canopy, snow and ice effects. It uses four soil layers of thicknesses 0, 30, 60, and 100 cm from the top down. The Betts-Miller-Janjic (BMJ) scheme was employed for the purposes of convective parameterization so as to provide better monsoon rainfall predictions^21,25,26^, since this method produced best predictions for the climate zones of India as well as inter annual variations in precipitations. During the course of simulation, the model results were archived every six hours.

#### Land use land cover (LULC) information

The default land use and cover data within the WRF model do not precisely represent our study period's actual land surface conditions in 2009 and 2014. As such, the Indian Remote Sensing (IRS) Satellite P6 Advanced Wide Field Sensor (AWiFS) data, which corresponds with MM5 & WRF frameworks at 30 arc-second resolutions, was extracted from the National Remote Sensing Centre online repository (http://bhuvan.nrsc.gov.in) and subsequently adopted to represent the actual land surface condition for 2009 and 2014.

#### Vegetation parameterization

Within the WRF model, vegetation phenology is represented by the green vegetation fraction (fg) as a dynamic tool. AVHRR satellite data with a monthly temporal resolution and spatial resolution of 0.144° spanning 1986 to1991 were employed to derive the current fg data. As such, the data does not take into consideration any more recent land changes or any weekly or bi-weekly vegetation impacts that resulted from extreme events. Simulations that represented the inter-annual variations in vegetation were developed based on a ten-day composite of vegetation fraction that was extracted from INSAT to produce a normalized difference vegetation index (NVDI).

The Indian geostationary satellite (INSAT3A) collates images of the surface of the continental (Asia) area of the Earth’s surface on a half-hour basis in a single snapshot at a spatial resolution of 1 km × 1 km. It is the only satellite that currently scans Asia with multi-spectral bands. The CCD payload has been purposely created to track snow coverage and vegetation throughout Asia at the available 1 km × 1 km resolution using three optical bands: red (0.62–0.68 m), near-infrared (0.77–0.86 m), and short-wave infrared (SWIR; 1.55–1.69 m). The NDVI obtained from INSAT at ten-day periods delivers a more reliable green vegetation fraction than the AVHRR-based climatology data that is employed in WRF^46,47^. For the estimation of the vegetative fraction the mosaic-pixel model was carried out in this study.

#### Verification approach

The rainfall output from WRF was validated with the observed Tropical Rainfall Measuring Mission (TRMM) daily rainfall data, which was gridded at 0.25° × 0.25°. The daily precipitation modeled within the simulation re-gridded at a resolution of 25 km × 25 km was contrasted with the equivalent Tropical Rainfall Measuring Mission (TRMM) rainfall data for 2014 and the IMD gridded rain-gauge data for the 2009 and 2014 simulations. The bias that various categories of rain rate contributed to the overall rain was also determined as a means of verifying the extent to which the model was deficient in terms of the spatiotemporal rainfall distribution. The daily temperature was also validated against data compiled by the ISRO meteorological station. The maximum and minimum monthly temperature was validated using the CRU monthly gridded data attained from CRU TS 3.21. The statistical metrics such as coefficient of determination (R^2^), Root Mean Square Estimation (RMSE), index of agreement, and BIAS were used to evaluate the model performance quantitatively for simulated rainfall. The BIAS measures the tendency to systematically overestimate or underestimate a parameter, RMSE gives a measure of absolute mean error, R^2^ provides the index of correlation with the increasing or decreasing trends in a given parameter with observations. Details about individual model evaluation statistics is described in “Model evaluation statistics” section.

### Decision support system agrotechnology transfer (DSSAT)

The Decision Support System for Agrotechnology Transfer (DSSAT) is a software framework for agricultural decision support. DSSAT comprises process-based crop simulation models for 47 types of crops^48^. It includes a database management system for soil, weather, crop management, and crop cultivar for crop growth simulation. DSSAT facilitates preparation of the input data, and includes application programs for seasonal, crop rotation, and spatial analysis. Its economic model computes gross margins based on harvested yield and by-products, the price of the harvested products, and input costs. For rice, the DSSAT has CERES (Crop Estimation through Resources and Environmental Synthesis) v3 module for rice crop yield simulation^15,49^. The CERES-Rice model^50^ is a process-based model of rice crops which simulates crop growth, development, and yield, taking into account the effects of soil water, weather, genetics, irrigation, planting, nitrogen, and carbon. The minimum inputs required for this model are daily weather, soil information, crop genetic coefficient, and management practices.

#### Weather data

The CERES-Rice model relies on minimum weather inputs, such as solar radiation, rainfall, and minimum and maximum temperature. WRF simulation output data for 2009 and 2014 were employed as the crop model input. NCO (net CDF Operators), CDO (Climate Data Operators), and NCL (NCAR Command Language) tools were employed to obtain the daily weather variables from the WRF outputs. Python programming was used to convert these outputs into a DSSAT weather file format.

#### Soil data

1:250,000 scale soil map available at the ICAR-National Bureau of Soil Survey and Land Use Planning (NBSS & LUP) was used to determine the precise taxonomical unit for Haryana and Punjab. NBSS & LUP publications related to the Haryana and Punjab soil series combined with the data compiled during field surveys were used to produce in-depth profile data for the taxonomical unit. The profile data was input into the DSSAT soil module, which assimilates data from four sub-modules: soil temperature, soil water, soil dynamics, and soil carbon and nitrogen. The soil module uses the information available to calculate the values that are absent from the available data. These values were stored in a DSSAT soil file format (Table S1–S5 in Supplementary Information Section 1). The soil file was assigned spatially to the study area through the use of a Python program.

#### Crop management

Crop management data were extracted from literature outlining standard agronomic practices from the state agricultural universities, Haryana Agricultural University (HAU), Hissar, and Punjab Agricultural University (PAU), Ludhiana^51,52^. The gathered parameters (Table S6 in Supplementary Information Section 1) included sowing depth; row spacing of plants; plant population; type, amount, and date of fertilizer application; depth of irrigation; type of irrigation; and irrigation schedules^53,54^. Even though there will be a difference in management practices by individual farmers, the majority of farmers' adoption was based on the practice of irrigation and nutrient applications as per the package of practices. However, it has not been considered the same for the entire study area since the soil characteristics varied, and that predictor was considered for simulations.

#### Yield assessment

Rice crop yield was simulated using the minimum data sets such as daily weather, soil information, crop genetic coefficient, and management practices within the DSSAT crop module^55^ (Table S7 in Supplementary Information Section 1). This yield was validated with district-wise observed yield from the Directorate of Economics and Statistics (DES), the mandated organization under the Ministry of Agriculture (MoA), Government of India (GOI) for collecting and reporting agricultural production data in India.

### Time series analysis of yield departure due to water stress

Detrending methods can be employed to determine climate-related yield as a means of extracting yield information. The ultimate objective of detrending techniques is to acquire a reliable annual record of yields that can be compared with the water balance data produced by the DSSAT CERES framework. The hypothesis asserts that any upward yield trend (a downward trend is unlikely) that results from the science and technological aspect should be removed from the data^56,57^. After the completion of the detrending process, the yield that was recorded in previous years will maintain predictive value in terms of the current season.

The commonly employed linear detrending approach was used in the current study to eliminate the trend aspect. Linear detrending involves fitting a straight line, T(t) = a0 + b0t, to the given time series. The subsequent straight-line detrending process eradicate the trend from the time series resulting in a zero-mean residue. Then, the detrended yield departure in combination with the yield anomaly, which was derived from the detrended yield and the average yield, was used to determine a reduction in yield that could be attributed to the water stress conditions. In DSSAT, water stress was imposed based on different irrigation levels and this was compared with the obtained results from the time series analysis.

**Figure 3:**
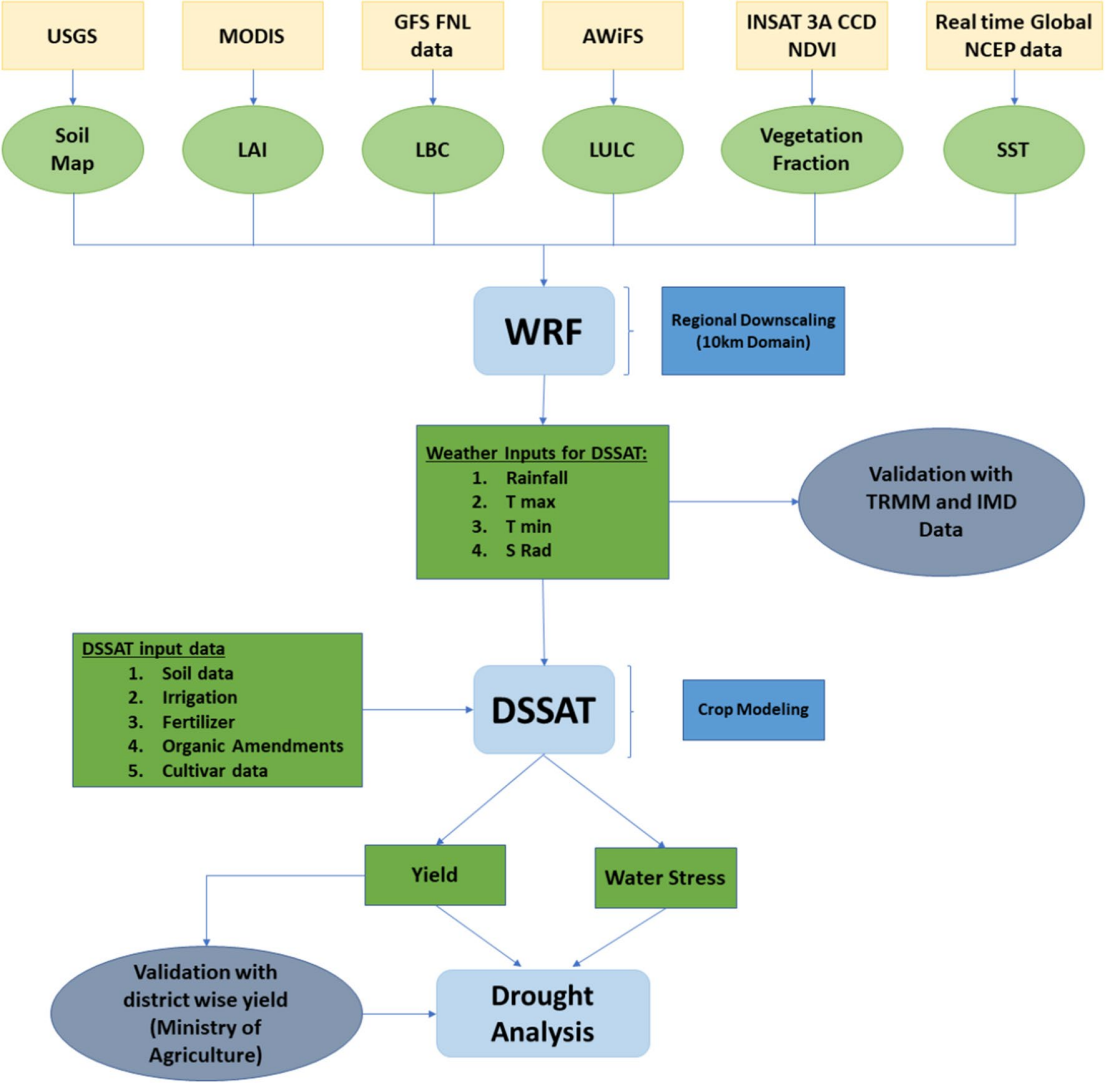
The overall methodology used in this study.

A detrended yield anomaly of the historical district-level yield data was used to analyze the water balance components of the DSSAT. Water balance component data can play a fundamental role in facilitating an understanding of the relationship between variation in weather conditions and a reduction in yield. DSSAT includes a variety of water balance elements, including evapotranspiration (ET), soil water availability, and potential evapotranspiration (PET) at daily intervals. Both ET and PET were calculated at three different stages of the rice crop (1–60, 60–120, and 120–146 days) to study the impact of water stress during these stages. The crop water stress was also estimated using PET and ET at 10-day intervals to understand the evolution of water stress in rice crop growing season. The overall methodology for the present study is shown in Fig. 3.

### Model evaluation statistics

Coefficient of determination (R^2^): Coefficient of determination describe the degree of collinearity between simulated (s) and observed (o) data. R^2^ describes the proportion of the variance in measured data explained by the model. R^2^ ranges from 0 to 1, with higher values indicating less error variance, and typically values greater than 0.5 are considered acceptable^58^.

Root Mean Square Error (RMSE): Several error indices are commonly used in model evaluation. These include mean absolute error (MAE), mean square error (MSE), and root mean square error (RMSE). These indices are valuable because they indicate error in the units (or squared units) of the constituent of interest, which aids in analysis of the results. RMSE, MAE, and MSE values of 0 indicate a perfect fit. RMSE (Eq. 1) values less than half the standard deviation of the observed data may be considered low and that either is appropriate for model evaluation. RMSE is a commonly used error-index statistic, and the lower the RMSE is, the better the model performance^59^.

1 $${\text{RMSE}}\, = \,\sqrt {\frac{1}{n}\mathop \sum \limits_{i = 1}^{n} \left( {o_{i} - s_{i} } \right)^{2} }$$

where, n represents the number of observations; o_i_ represents the ith observed value and s_i_ represents the ith model simulated value.

The bias (BIAS):- is the deviation of data being evaluated, expressed as a scalar unit. It measures the average tendency of the simulated data to be larger or smaller than their observed counterparts. The optimal value of BIAS is 0.0, with low-magnitude values indicating accurate model simulation. Positive values indicate model underestimation bias, and negative values indicate model overestimation bias^60^.

Index of agreement (d): The index of agreement (d) was developed by Willmott^61^ as a standardized measure of the degree of model simulation error (Eq. 2) and varies between 0 and 1. A computed value of 1 indicates a perfect agreement between the measured and predicted values, and 0 indicates no agreement at all^61^.

2 $${\text{d}}\, = \,1 - \frac{{\mathop \sum \nolimits_{i = 1}^{n} \left( {o_{i} - s_{i} } \right)^{2} }}{{\mathop \sum \nolimits_{i = 1}^{n} \left( {\left| {s_{i} - \hat{o}} \right| + \left| {o_{i} - \hat{o}} \right|} \right)^{2} }}$$

where, n represents the number of observations, o_i_ represents the ith observed value, s_i_ represents the ith model simulated value and ô mean of the observed values for the entire period.

Further details about multiple performance metrics are available in the following papers^62,63^.

### Ethics approval

The work in this paper was carried out by Mr. T Rajasivaranjan under the guidance of Dr. N R Patel, Scientist-F, Agriculture & Soils Department, IIRS, Dehradun, and in partial fulfilment for the award of degree of Master of Technology in Remote sensing & GIS.

## Results

### Evaluation of WRF simulations

#### Rainfall variability

**Figure 4:**
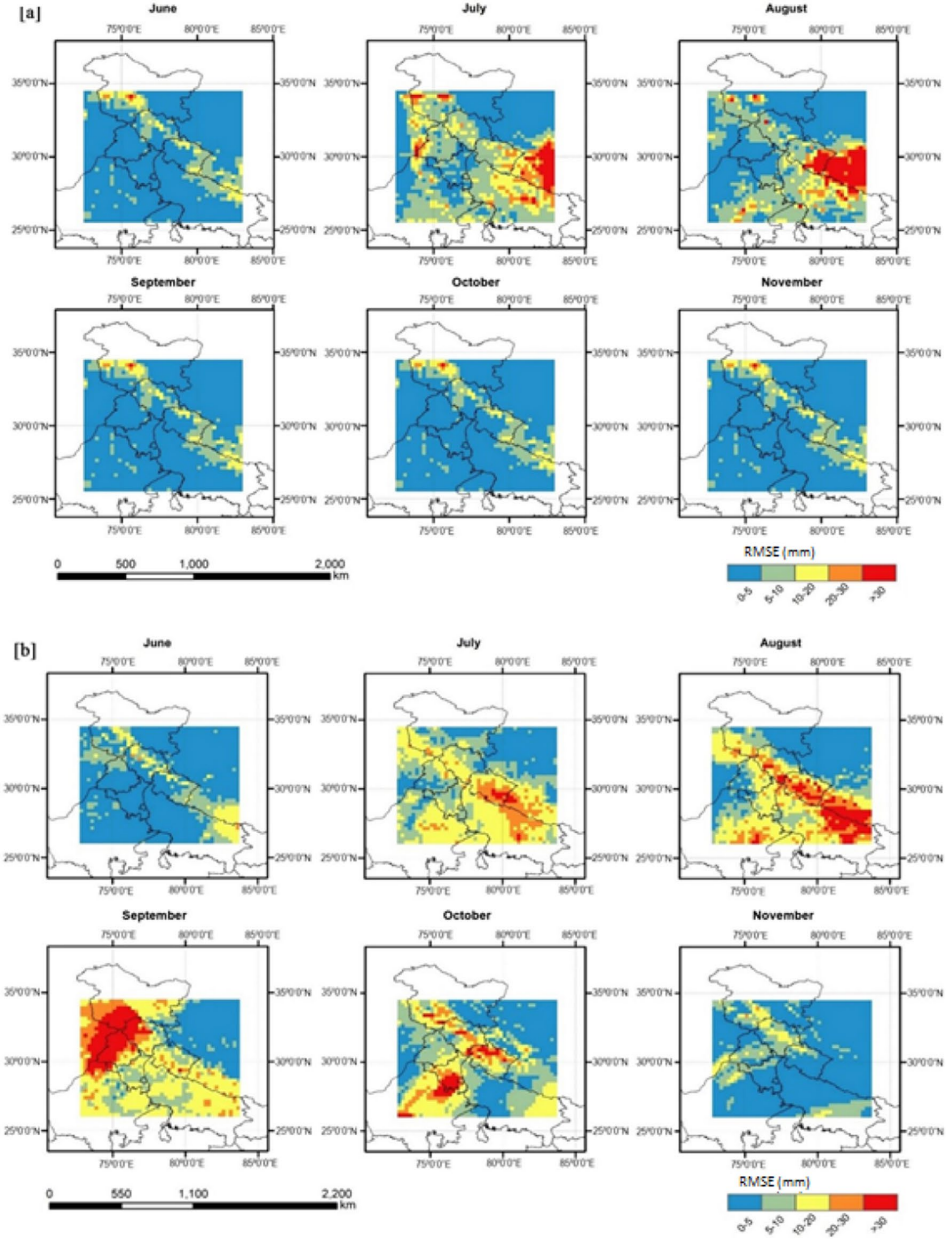
Spatiotemporal validation of WRF monthly rainfall simulations against TRMM based on RMSE (mm) for the months from Jun to Nov during (**a**) 2009; (**b**) 2014 (Map created at IIRS—https://www.iirs.gov.in/inhouselaboratories).

**Figure 5:**
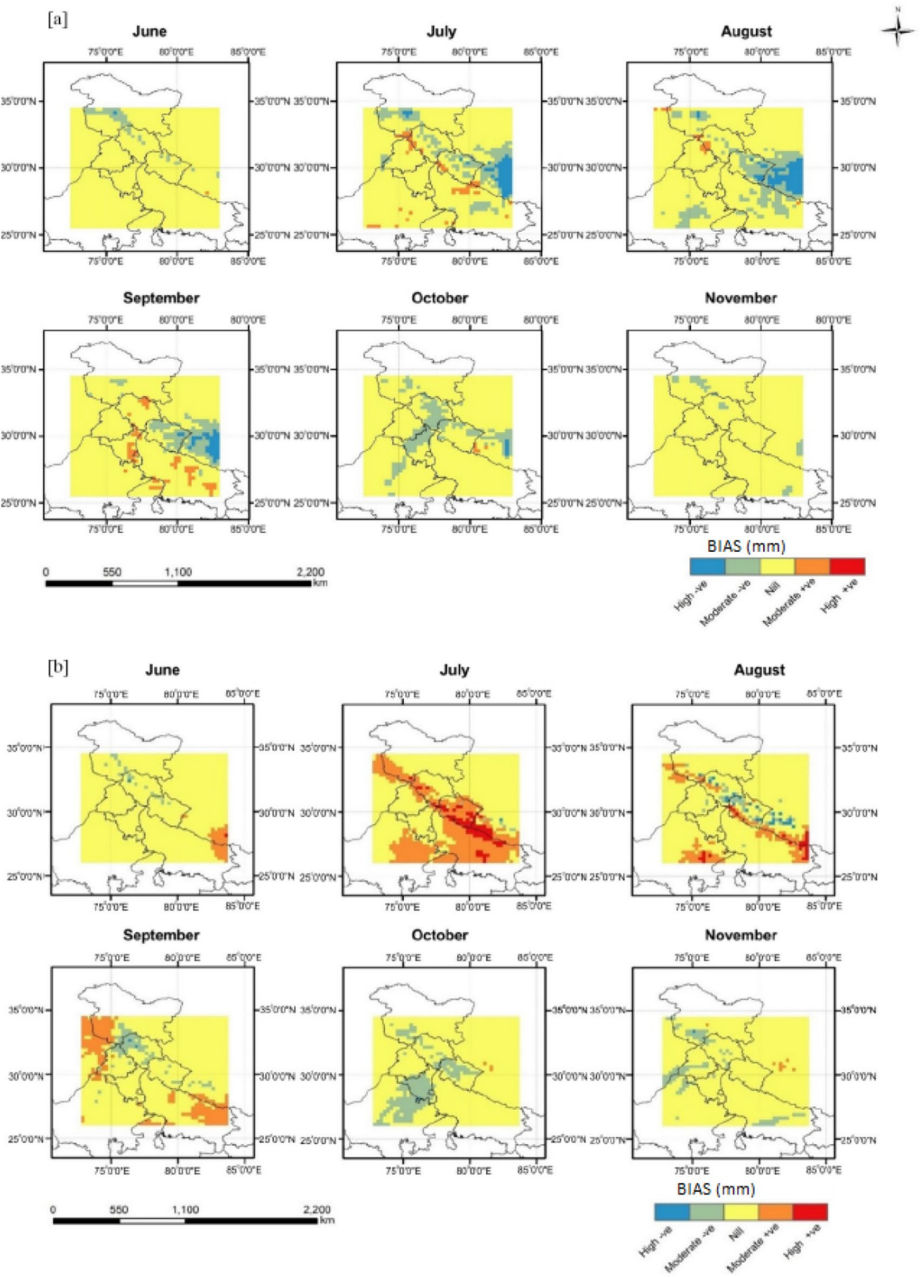
Spatiotemporal pattern of rainfall bias (**a**) 2009; (**b**) 2014 (Map created at IIRS—https://www.iirs.gov.in/inhouselaboratories).

The rainfall output from the WRF model was validated with the observed TRMM rainfall time series, at the spatial resolution of 0.25° × 0.25°. Figure 4 represents the spatiotemporal performance of WRF rainfall simulations based on RMSE. The difference between the WRF simulation and TRMM time series was high during July, August, September, and October. Although the model could capture the spatial rainfall distribution, it underestimated the rainfall over the eastern Himalayas region in almost all months. The model also significantly underestimated rainfall during September (RMSE ≥ 30 mm), indicating the model’s inability to capture the torrential rain in the Kashmir valley. The monthly averaged bias was further analyzed to study the performance of the WRF model (Fig. 5). Accordingly, monthly rainfall for July (October) shows a positive (negative) bias over the central and Himalayan (western) regions, indicating the under (over)-estimation of our WRF model simulations.

**Figure 6:**
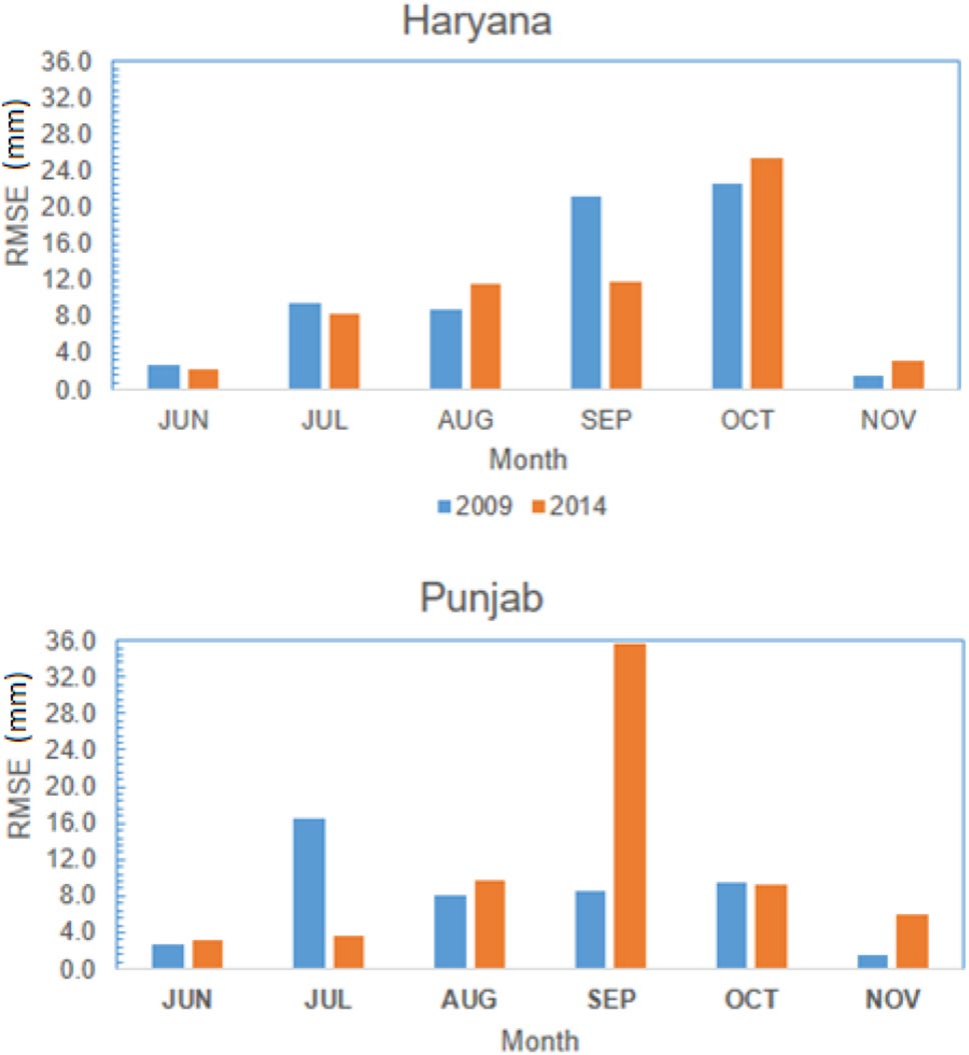
Regional validation of WRF monthly rainfall simulations against TRMM across Punjab and Haryana based on average RMSE values for each month from Jun to Nov during 2009 and 2014.

**Figure 7:**
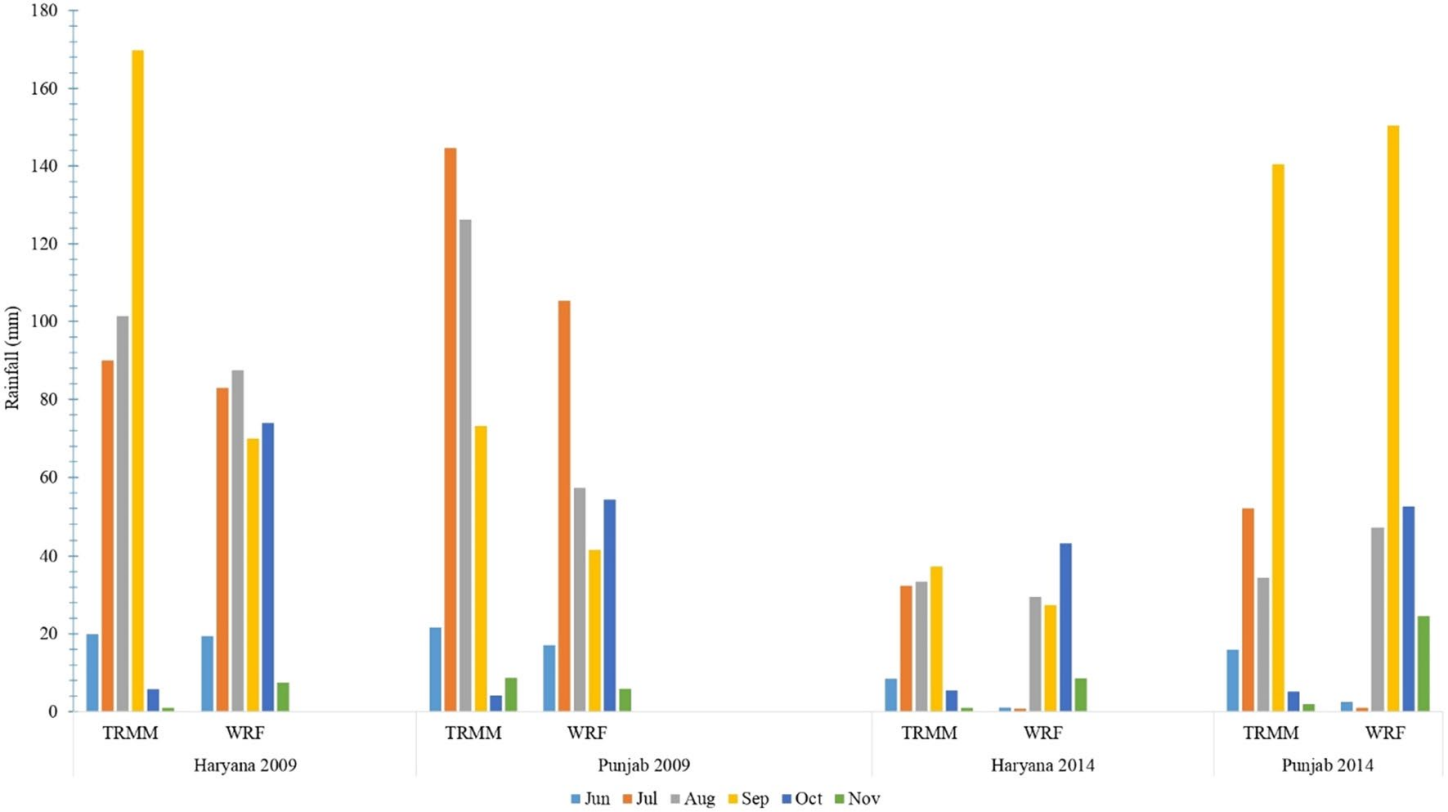
Comparison between simulated and observed monthly rainfall time series provided by the WRF model and the TRMM dataset, respectively, for Jun-Nov in Haryana and Punjab during 2009 and 2014.

During the drought-affected year 2009, the higher RMSE values were seen during July in Punjab, while during October and September in Haryana (Fig. 6). However, the RMSE values over the Punjab (Haryana) region were less than 10 mm in most monsoonal months, except September (October), through the year 2014 (Fig. 6). The WRF model considerably underestimated monthly rainfall across both Punjab and Haryana regions during October 2009 and 2014 (Fig. 7). In general, the WRF model underestimated the peak rainfall phase during Jun-Aug across the Punjab (Haryana) region during 2009^11^.

Average RMSE, bias, and agreement index values between the WRF simulated and the TRMM observed daily rainfall time series across the Punjab and Haryana regions are given in Table 1. The results show 9.4 (20.1) and 10.2 (16.3) mm variations in daily rainfall over the Punjab and Haryana regions during 2009 and 2014, respectively. The range of agreement index between the simulated and observed daily rainfall across Punjab and Haryana was 74.2–81.7% (Table 1). However, the negative bias values indicate that the WRF model generally underestimated daily rainfall over both study areas (Punjab and Haryana) during the drought-affected (2009) and (2014) years. Similarly, previous studies by Hong et al*.*,^27^ and Ma et al*.*,^43^ corroborate such findings.

**Table 1:** Statistical analysis of the WRF model’s performance in simulating daily rainfall over the Punjab and Haryana regions during 2009 and 2014, compared to the observed daily rainfall time series obtained from the TRMM product.

Statistics	2009		2014	
	Punjab	Haryana	Punjab	Haryana
RMSE (mm)	9.4	10.2	20.1	16.3
Agreement index (%)	81.7	79.4	77.6	74.2
Bias (mm)	− 17.2	− 10.1	− 20.7	− 13.5

According to the TRMM observed rainfall time series, the maximum rainfall was seen in the eastern zone in July and August (Supplementary Figures S1 and S2). However, there was a rainfall deficit in June, July, and August in India’s northwest area. A rise in the amount of rainfall throughout the Himalayas’ northern regions was seen in September. However, the variation in rainfall was marginally underestimated by the WRF model in comparison to the TRMM rainfall data (Supplementary Figures S3 and S4). An overestimation of rainfall in the eastern Himalayan area was also found during nearly all the WRF simulation months. Overall, the WRF model accurately simulated the rainfall variability in India’s eastern and arid and semi-arid northwestern regions. It implies that the northward circulation of the monsoon convection was accurately represented within the WRF model.

#### Evaluation of temperature simulations

The WRF model output was validated using the mean daily maximum and minimum temperatures extracted from nine ISRO meteorological stations, as these data sets are commonly accessible for validation studies. The error statistics and verification measurements for the maximum, minimum, and average temperatures are presented in Table 2.

**Table 2:** Error statistics of daily mean, minimum and maximum temperatures simulated by the WRF model in comparison with data recorded at nine ISRO meteorological stations.

Station name	RMSE (°C)			Agreement Index (%)		
	T_mean_	T_min_	T_max_	T_mean_	T_min_	T_max_
RS.SF, PAU. Kapurthala	2.54	3.33	6.75	85.58	80.47	52.12
KVK, Bahowal. Hoshiarpur	2.96	3.97	6.93	37.25	41.23	14.41
RRS, Kandi Area. Balachaur. Nawan	2.34	3.53	6.92	85.58	77.44	50.46
KVK, Langroya. NawanShahar	2.27	3.47	6.80	87.67	77.45	55.21
Agromet dept., PAU campus. Ludhiana	4.44	4.67	8.37	64.81	67.56	39.32
KVK, PAU farm, Samrala. Ludhiana	3.08	2.81	6.62	43.27	52.12	32.13
KVK, Mallewal farm. Ferozepur	3.05	3.38	6.10	81.69	81.30	59.20
RRS-PAU farm, Abohar. Ferozpur	2.86	3.80	5.74	83.94	76.88	65.95
KVK, VPO Goniana. Muktsar	2.94	2.42	7.39	86.58	91.54	54.20
Overall average	2.94	3.49	6.85	72.93	71.78	47.00

**Figure 8:**
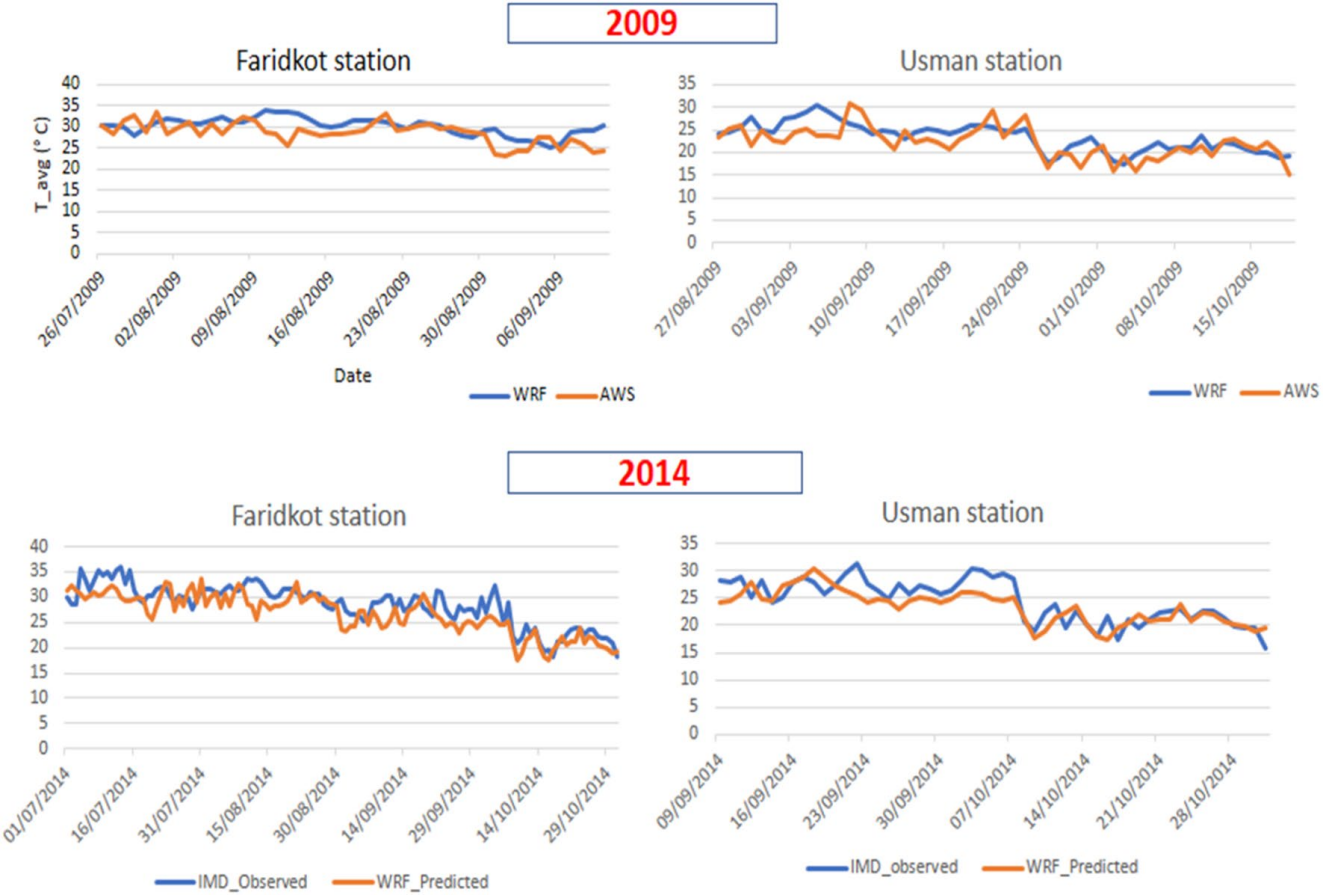
Simulated and observed mean daily temperature (°C) timeseries obtained from the WRF model and ISRO, respectively, at the Faridkot and Usman hydrometeorological stations during 2009 and 2014.

An RMSE error between 2.3 and 3.1 °C was observed at the majority of stations. However, the RMSE was in a higher order for the maximum temperature across all the stations. A higher agreement index was seen for the average temperature, followed by the minimum and maximum temperature. The simulated results show a slight variability in observed temperature (Fig. 8).

### Yield simulation of rice using DSSAT crop simulation model

**Figure 9:**
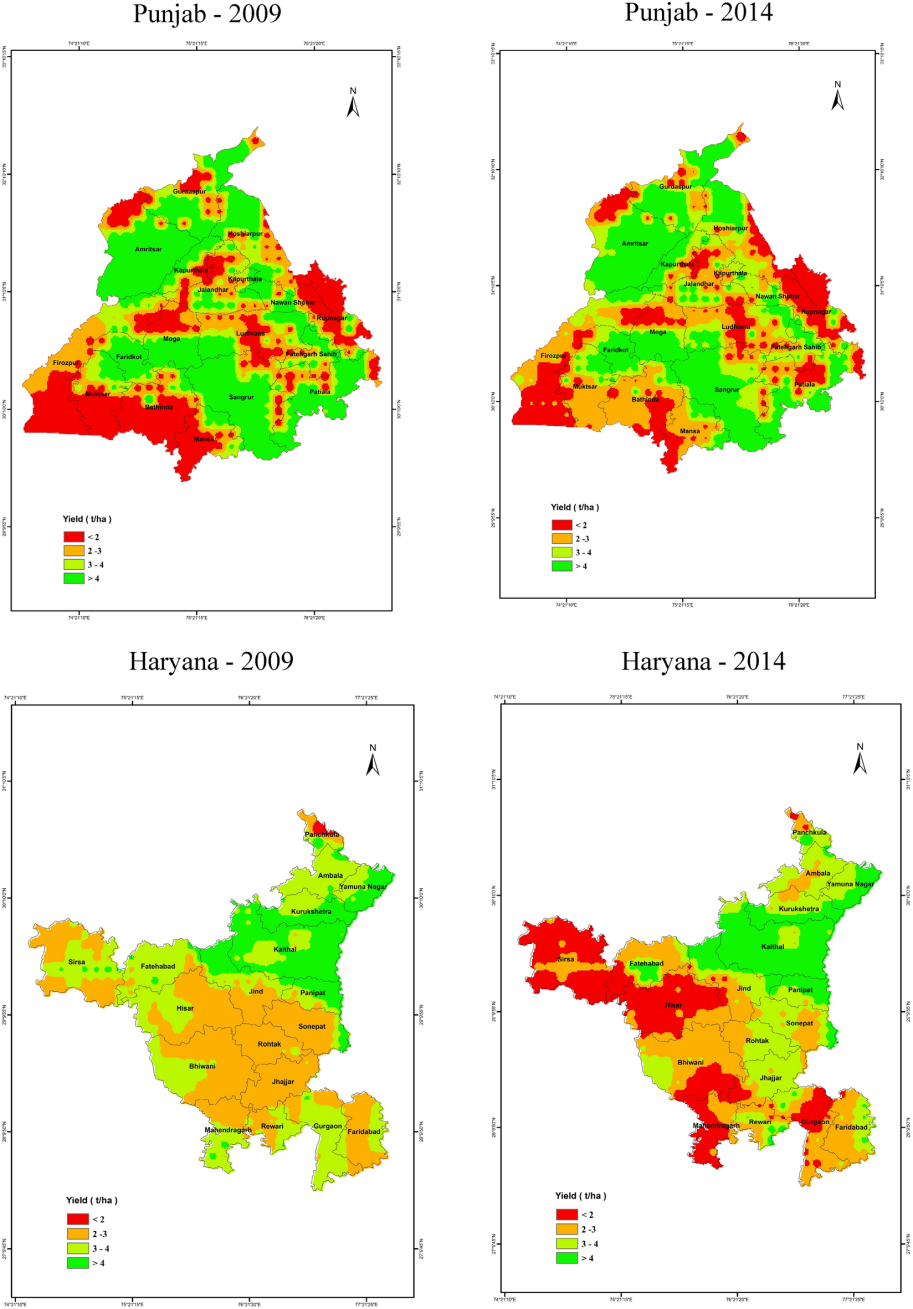
Spatial distribution of rice yield (t/ha) in the Punjab and Haryana regions for 2009 and 2014 years simulated by the DSSAT crop model (Map created at IIRS—https://www.iirs.gov.in/inhouselaboratories).

**Figure 10:**
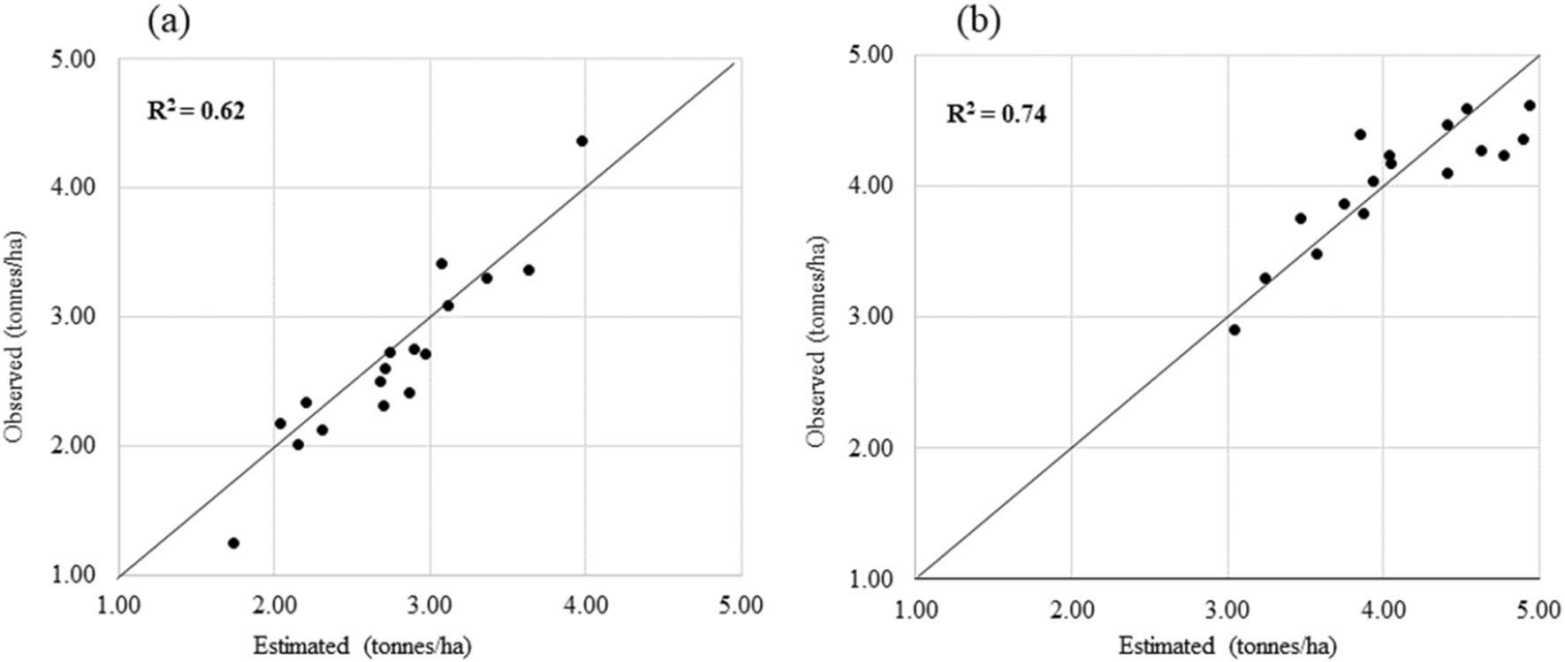
Relationships between simulated and observed rice yield in (**a**) Punjab and (**b**) Haryana, during 2009. Dots refer to district level spatial aggregation (average) of rice yields estimated based on the DSSAT simulations.

The simulated rice yields for 2009 and 2014 over Haryana and Punjab are presented in Fig. 9. These simulated yields were comparable to the range of yields typically reported by the Government of India (GOI). The rice yield estimated in 2014 was higher than that of 2009 in most of Haryana's districts. But the yield was lower in 2014 than in 2009 in districts such as Fatehabad and Hisar. The yields in the Gurdaspur, Kapurthala, Amritsar, Jalandhar, and Hoshiarpur districts of Punjab were higher in 2014 than in 2009, and there was a lower yield in other districts. The DSSAT crop simulation adequately modeled Haryana and Punjab's crop yields with sufficient R^2^ values of 0.62 and 0.74, respectively (Fig. 10).

#### Prediction of yield loss during the 2009 drought

**Figure 11:**
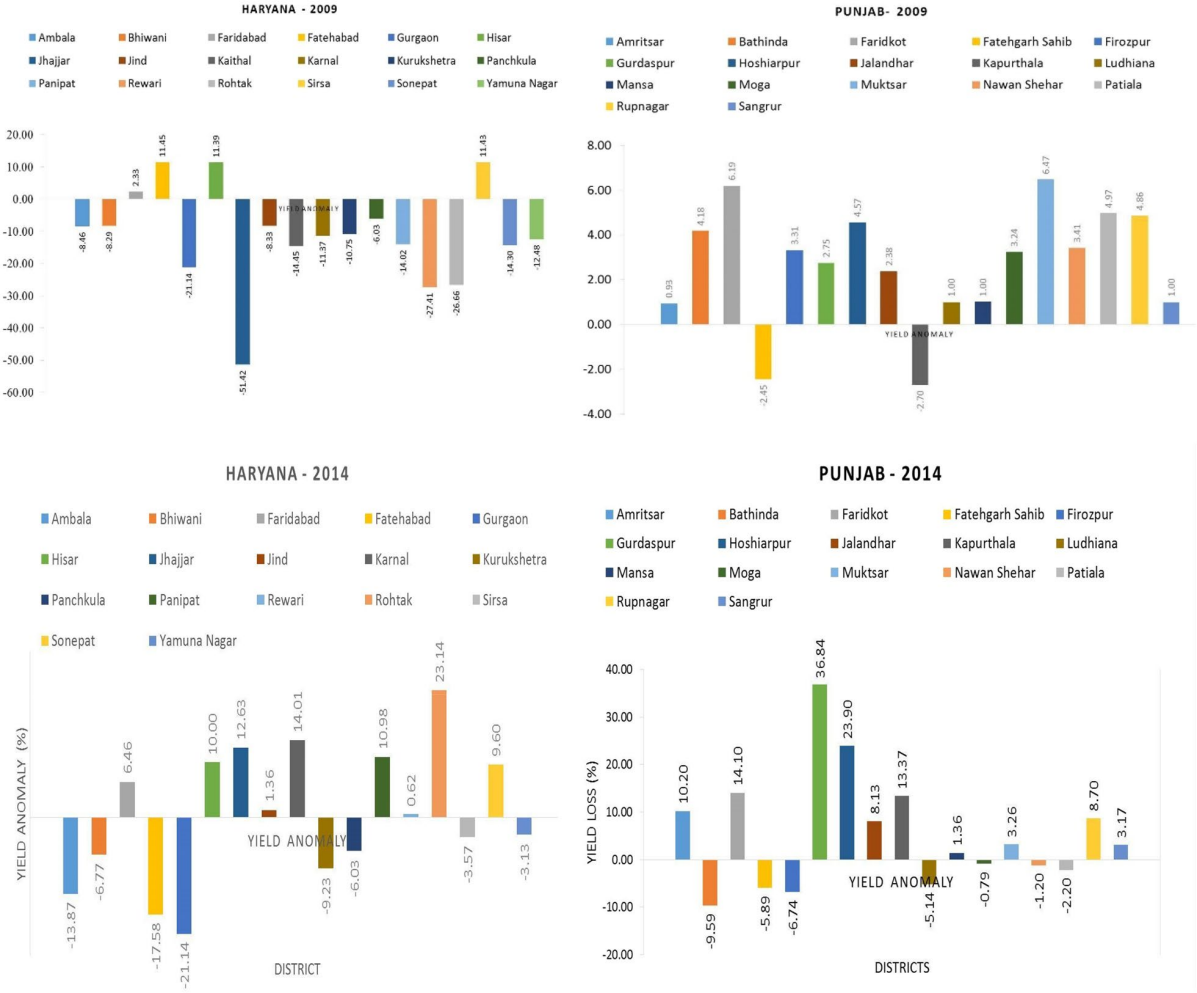
Annual anomaly of rice productivity (%) in different districts of Punjab and Haryana for 2009 and 2014.

The anomaly of crop yield based on the mean and detrended yield for rice productivity was calculated to estimate the yield departure due to climatic variability. The relative detrended yield is the departure of yield values from the time-trend, assumed to take into account the technology and management component of yield. Figure 11 shows the district-wise rice yield anomaly based on average yield and detrended yield over Haryana and Punjab respectively during 2009. From the figures, it is clear that the severity of the drought was comparatively higher in Haryana than Punjab during 2009. Almost all districts in Haryana were affected and in particular, districts such as Jhajjar, Rewari, Rohtak, and Gurgaon experienced the deleterious effects of drought conditions, which reduced the crop yield by around 51, 27, 26, and 22 percent, respectively. Although the water stress impact was severe, some of the districts, such as Fatehabad, Faridabad, Hisar, and Sirsa, showed a positive yield anomaly, which reflects an increase in rice productivity. In Punjab, the scenario was different, and the crop productivity was surprisingly high during drought years and almost all districts recorded a higher crop productivity.

#### Crop water stress and yield relation

**Figure 12:**
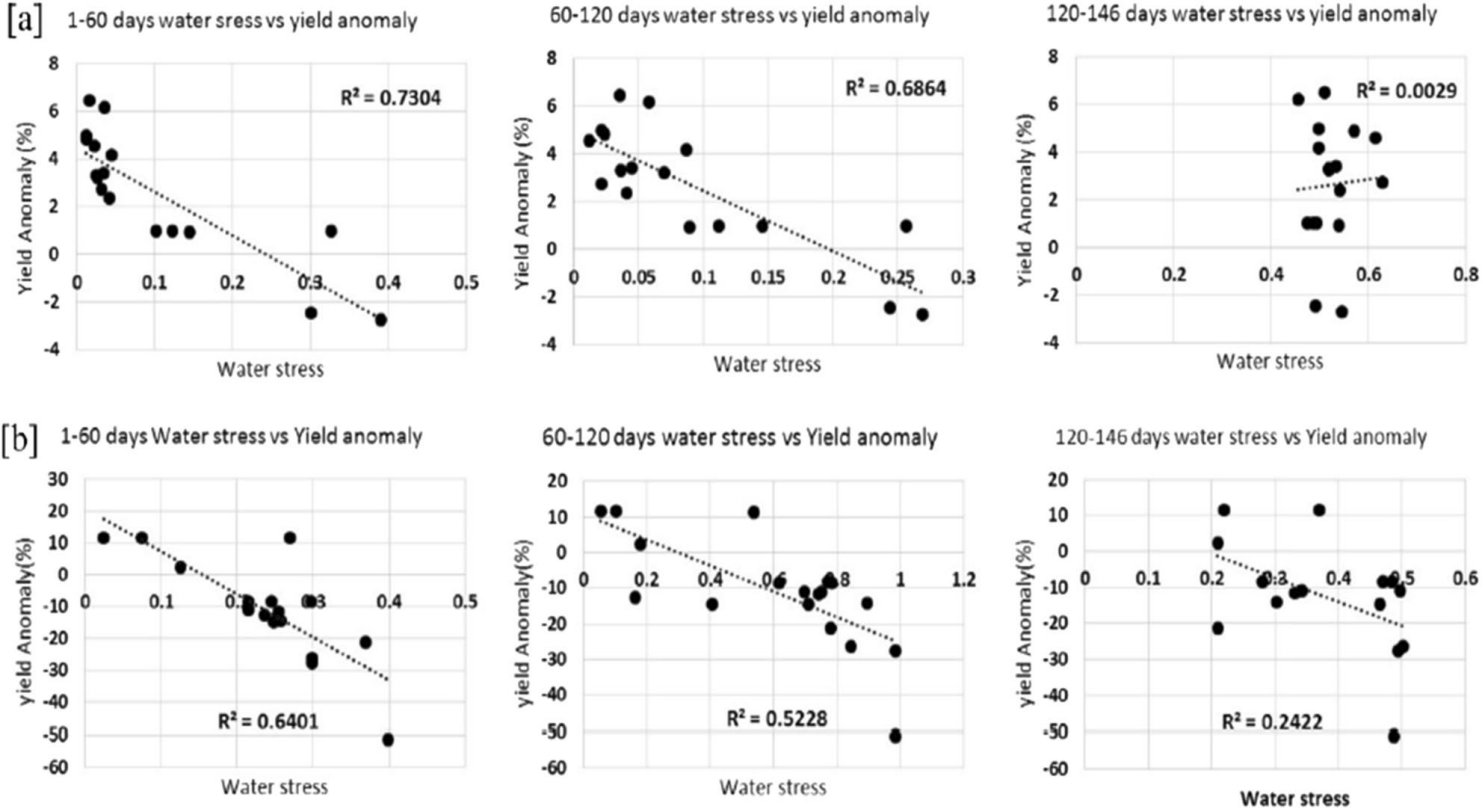
The relationships between annual rice yield anomaly in 2009 and the simulated rice crop water stress factor at the vegetative (1–60 days), reproductive (60–120 days), and maturity (120–146 days) stages throughout (**a**) Punjab and (**b**) Haryana.

Figure 12 depicts the yield anomaly in contrast to the forecasted crop water stress factor at the vegetative stage (1–60 days), reproductive (60–120 days), and maturity (120–146 days). The outcomes reveal a statistically significant (*p* < 0.05) relationship between water stress in the vegetative growth stage (Days 1–60) and the reproductive growth stage (Days 60–120) and the yield anomaly of rice observed in 2009. The correlation between the crop water stress factor and the rice yield revealed an R^2^ value of 0.64 and 0.52 for the vegetative and reproductive stage of rice in Haryana and 0.73 and 0.68 for Punjab (Fig. 12). However, this study revealed no correlation between crop water stress and the yield during the maturity stage in Haryana and Punjab.

#### Spatiotemporal pattern of 10-day rice crop water stress factor in 2009

**Figure 13:**
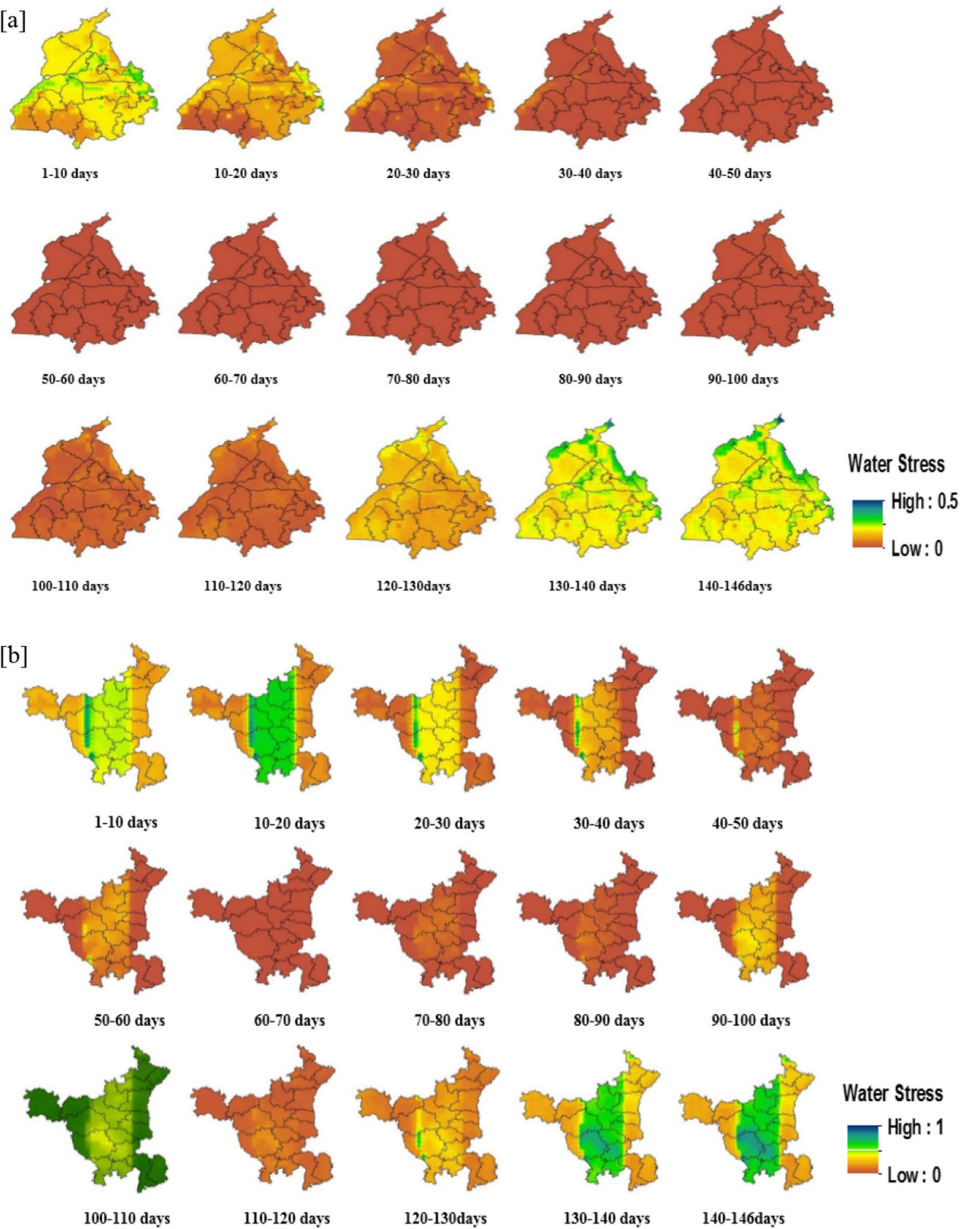
Spatio-temporal distribution maps of rice crop water stress actor at 10-day interval for (**a**) Punjab and (**b**) Haryana during 2009 (Map created at IIRS—https://www.iirs.gov.in/inhouselaboratories).

The potential (PET) and total (ET) evapotranspiration rates during all three different stages of rice crop growth throughout Punjab and Haryana during 2009 are given in Supplementary Figures S5. Such DSSAT-projected evapotranspiration closely followed the spatial WRF rainfall simulations, providing valuable information for estimating the crop water stress and yield of rice in Punjab and Haryana. Accordingly, the spatio-temporal pattern maps of rice crop water stress factor at the 10-day interval for Punjab and Haryana during 2009 are illustrated in Fig. 13. Based on these maps, Haryana generally experienced more severe rice crop water stress factor (0–1) than Punjab (0–0.5) in 2009.

Due to the delayed onset of the monsoon, the rice crop water stress evolves gradually in the initial stages, and then declines in the central districts of Haryana. The central parts of Haryana also show a sudden increase in water stress and drought severity during the rice reproductive stages (60–120 days) due to the deficient rainfall and the high evaporative demand in August 2009. Districts such as Jhajjar, Rohtak, Rewari, Gurgaon, Bhiwani, Hisar, and Sonepat show severe (moderate) rice crop water stress factors during 10–20 (20–30, 30–40, and 90–100) days. Moreover, the annual rice yield anomalies of those districts show a drastic negative change that reflects the water stress during vegetative and reproductive stages with a profound impact on rice productivity. The spatial pattern shows minimal stress in the initial stages with water stress values ranging from 0 to 0.2 and indicates that there was no water stress condition in Punjab during 2009. Even though the spatio-temporal stress is seen in Punjab, it did not impact the rice yield since most of the regions is irrigated with groundwater. Although the crop water stress condition during 2009 in Punjab did not have any direct impact on its rice productivity, it affected the overall economy of the state due to the increased cost of diesel and the decline in the groundwater table for succeeding wheat crops.

The annual yield anomaly and spatially averaged water stress at different rice crop stages were compared to study the severity of water stress in Haryana’s drought-affected districts. The plot in Supplementary Figure S6 shows the comparison of crop water stress at vegetative (1–10 days, 10–20 days, 20–30 days, and 30–40 days) and reproductive (90–100 days and 100–110 days) stages against yield reduction for rice in Haryana during 2009. The rice crop water stress at 90–100 and 100–110 days (i.e., flowering to maturity stage) show a high degree of fit with the annual rice yield anomalies in the Haryana region in 2009. This anomaly implies that the high sensitivity of rice to water stress with any intensity (mild or severe) during the reproductive stage has the most severe and devastating effects on yield. The initial stage water stress shows a moderate association with annual yield anomalies, reflecting a moderate impact on rice production. On the other hand, there was a lack of significant correlations between the water stress during the maturity stage and variations in annual rice yield anomalies of drought-affected districts in Punjab and Haryana in 2009.

## Discussion

### Evaluation of WRF simulations

The rainfall output from WRF was validated with the observed TRMM daily rainfall data, and maximum and minimum temperature data was validated with IMD observed data. The WRF regional climate model was used to produce a local climate simulation of the Indian summer monsoons observed in North India in 2009 and 2014. In the year 2009, India received just 77 percent of its annual rainfall^64^ and the country experienced a severe drought. Unlike 2009, monsoon progressed slowly in 2014, producing below-average rainfall until mid-July, rainfall exceeding normal conditions for July and early August, then rainfall dropping again below normal and ultimately reaching the peak (above normal conditions) during late August and early September^7^. The rainfall in 2014 was 12 percent below the monsoon average.

In general, the WRF model accurately simulated the minimum and maximum rainfall in India’s eastern and arid and semi-arid northwestern regions. It implies that the northward circulation of the monsoon convection was accurately represented within the model. In some regions, increase in rainfall was observed and this increase in precipitation could be attributed to the heavier rainfall in the Kashmir valley. As discussed in results, in some of the months the model overestimated the precipitation and in some months it under estimated the same. This kind of varied performance of the WRF model was also observed and reported^30–32^.

Precipitation simulation by regional climate models (RCM) is highly sensitive to the representation of convection which is an important physical process underlying precipitation. Convection in climate models is formulated mainly through implicit convective (cumulus) parameterization (CP) schemes and explicit cloud microphysics (MP) schemes. While the cumulus schemes compute the effects of sub-grid scale convective processes on the grid scale variables and removes the convective instability, the microphysics explicitly compute the small-scale physical processes governing the cloud formation, growth, and dissipation that are responsible for moist convection. At grid resolutions of ≥ 10 km where most of the RCMs operate mainly convective parameterization is used. The previous RCM studies of Indian Monsoon rainfall climatology^21,25,65^ showed that WRF simulated precipitation was sensitive to the convective parameterization. All these studies confirm that while Kain-Fritsch (KF) scheme produces moist bias, the Grell-Devenyi (GD) gives dry bias and Betts-Miller-Jangic (BMJ) scheme realistically simulated the monsoon rainfall over various climate zones of India. The study by Srinivas et al*.*^25^ also showed that the BMJ could simulate the inter-annual variation of monsoon rainfall in good fidelity with observations, however with slightly lesser rainfall during El Nino years and slightly higher rainfall for La Nina years. The varied performance of WRF in the present study for 2009 and 2014 years corroborates with the previous studies^25,65^ the slight dry bias in 2014 could be due to the convection scheme (BMJ) used in the present study, which has a slight dry bias for deep convective systems^21^.

#### Evaluation of monthly precipitation

This underestimation is due to high orographic variation in the land surface, which affects the region’s boundary condition. Moreover, TRMM data sets are derived from active precipitation radar (PR), passive microwave radiometers (MWR), Microwave Imager (TMI), and infrared radiometer (IR) sensors using universal sensor algorithm for the whole globe without considering the local condition. The extreme rainfall events in some parts of India could be due to multiscale interaction with local topography^66,67^. The high RMSE for rainfall was likely due to the coarse resolution (10-km) model’s limitation to resolve the mesoscale convective organization and applying the BMJ convection scheme for high rainfall simulation^25^.

#### Overall performance of WRF rainfall simulations

This negative bias is due to high orographic topography and the model deficiency at 10 km resolution in simulating the mesoscale effects on convection and associated heavy rainfall. Although the model captures spatial structure, overall, the amplitude of variability in rainfall is slightly underestimated when compared to TRMM observation. However, the WRF simulated rainfall overestimates monthly rainfall over the eastern Himalayas region. The results on error statistics and verification measures of WRF showed 10.2 mm variation in daily rainfall over Haryana and 9.4 mm variation over Punjab with an agreement index of 79 and 81 percent respectively for the year 2009 (Table 1) and almost similar results were observed for 2014 also. The results depicted that the downscaled products of WRF captured well the rainfall in many of the regions in both the states, even though there are small overestimation or underestimate for few regions compared to the observed rainfall data. The model evaluation statistics confirmed that this dynamically downscaled model WRF makes it suitable to simulate rainfall data. The overestimation of rainfall by WRF might be due to increased pressure gradients and enhances the convection in the upper streams and associated rainfall^25,68,69^. Some of the earlier studies by Hong et al*.*^27^ and Ma et al*.*^43^ corroborate these findings.

### Temperature

For temperature data, these nine ISRO meteorological stations (Table 2) were selected based on the data availability and ease of getting the data, and data quality. However, in the past, several studies have used fewer meteorological stations in data-scarce areas^70^. The model underestimated the frequency of high mean temperature during October. It indicates that there was a cold bias in the maximum temperature. Shah and Mishra^71^ also reported the cold bias in maximum temperature and warm bias in minimum temperature. Such bias arises from the boundary layer physics due to the stronger turbulent mixing of heat^32^.

### Yield simulation of rice using DSSAT crop simulation model

The simulated crop yield by DSSAT is compared with the district-level yield statistics derived from the GOI, Ministry of Agriculture data. The DSSAT crop simulation adequately modeled Haryana and Punjab's crop yields except for few districts. Among both the years, 2009 showed lower yields in both the regions and it is clear that the severity of the drought was comparatively higher in Haryana than Punjab during 2009. Lower yields in 2009 in Haryana are consistent with the severe drought caused by a delayed monsoon^72,73^ as reported earlier. However, in Punjab, the scenario was different, and the crop productivity was surprisingly high during drought years. The productivity of rice is least affected by water stress due to an extensive practice of growing drought-tolerant varieties and because of assured irrigation through pumping of groundwater to save paddy crops. In addition to that, the increase in the number of sunny days with adequate water during drought years creates a favorable condition for rice production. Although the drought during 2009 did not have any direct impact on crop production, it affected the overall economy of the state due to the increased cost of diesel and the decline in the groundwater table for succeeding crops^73^.

### Crop water stress and yield relation

The results revealed that a statistically significant relationship between water stress in the vegetative growth stage (Days 1–60) and the reproductive growth stage (Days 60–120) and the yield anomaly of rice observed in 2009 and however no significant correlation was observed between the water stress and maturity stage (Fig. 12). As reported earlier, rice crop facing water stress during maturity did not have any significant effect on the yield. Furthermore, the development of water stress conditions during the vegetative and flowering stages of rice crop growth result in higher yield losses than those observed during the latter stages of growth i.e., at maturity stage^74–76^. The reduction in yield resulting from water stress was less in Punjab than that observed in Haryana and indicates that water stress does not significantly impact rice crops in Punjab as it does in Haryana. It is worth observing that the water stress that was seen during the vegetative stage of the crop growth led to a significant reduction in the rice yield observed in Haryana compared to the water stress observed during the reproductive phase. The destructive implications of water stress of even lower magnitude during the vegetative stage of the rice crop were ascribed to the seedlings' failure to establish root due to the delayed onset of the monsoon^74^.

## Assumptions, drawbacks and future work

This study assumes nine ISRO meteorological stations are sufficient in data scares regions^70^. However, the results could be subjective on the number of stations used. Also, in this study, two years of drought patterns were assumed to be representative of the drought in the regions. The results in this study could be subjective to the drought patterns chosen. Using multiple years of drought patterns can bring more insight into drought patterns, predictions, and their impact on crop yield. These are differed for future studies.

Additionally, this study opens up several avenues for future work : comparison between yields simulated by DSSAT run with reference meteorological data vs. those obtained using downscaled data from WRF vs. yields observations; utilizing several crop models (e.g., Info Crop) with the downscaled climate data. Similarly, outputs from crop simulation models such as DSSAT, Infocrop, APSIM, etc., based on the inputs from WRF, can be compared with other downscaling models and other sources of climatic data. Those outputs will help to get great insight into the effectiveness of WRF in downscaling compared to other forecasting models and the reliability of the coupled crop simulation outputs.

## Conclusions

The current study attempted to develop an approach for drought monitoring and yield loss assessment by integrating geospatial system, climate, and crop simulation modeling at regional scales, by providing inputs from WRF into DSSAT. Results suggest that crop model DSSAT combined with WRF regional climate model output at 10 km can capture the evolution of drought and its impact on crop yield in a satisfactory manner. The WRF model performance after the introduction of improved land surface conditions extracted from the satellite data was assessed, and the outputs for the minimum and maximum rainfall and average temperature were determined to be satisfactory. The downscaled high-resolution data extracted from the WRF model was input into the crop simulation model (DSSAT) to provide insights into the drought-related factors that impacted crop production. A detrended yield anomaly of the historical district-level yield data was used to analyze the water balance components of the DSSAT.

In addition, simulated rice productivity was used to validate the district-level yield data. The results also revealed that rice crop vegetative and reproductive stages are more sensitive to water stress. The seasonal scale WRF simulations of rainfall using the current land use and land cover and vegetation fraction derived from the Indian Remote Sensing satellite over North India agreed reasonably with the TRMM rainfall estimates. The DSSAT-projected evapotranspiration closely followed the spatial WRF rainfall, thus providing valuable input in crop water stress and crop yield estimation. The WRF-DSSAT projections of yield anomaly followed the distinct rainfall spells and the water stress during different crop stages. This study clearly shows that the crop and regional climate model, combined with GIS and Remote Sensing, can practically measure the impact of crop water stress in terms of crop yield at a regional scale and help advocate suitable drought management programs. The reanalysis of data sets using WRF-DSSAT projections helps in the early prediction of crop water stress and its impact on crop yield at the regional scale to facilitate devising suitable drought management options to sustain rice productivity. This integrated approach can be used by downscaling global climate model outputs with WRF for an advance prediction of water stress and assessing their impacts on crop yields. Our approach facilitates devising strategies for mitigating the effects of water stress. The assumptions, drawbacks and future work in this study are discussed.

## Supplementary Information

Supplementary Information.
