# Supplementary material for: Integrated use of regional weather forecasting and crop modeling for water stress assessment on rice yield
Source: Sci Rep. 2022 Oct 10;12:16985. doi: 10.1038/s41598-022-19750-z (PMC9551056; doi:10.1038/s41598-022-19750-z)
Supplement: Supplementary file 1 — Supplementary Information. [file 41598_2022_19750_MOESM1_ESM.docx]

Integrated use of Regional Weather Forecasting and Crop Modeling for Water Stress Assessment on rice yield

(Supplementary Information)

T Rajasivaranjan^1^, Aavudai Anandhi^2^, N R Patel^1^, Masoud Irannezhad^3^, C V Srinivas^4^, Kumar Veluswamy^5*^, U Surendran^6^ and P Raja^7^

^1^Indian Institute of Remote Sensing, Dehradun-248001, India

^2^Biological Systems Engineering, Florida Agricultural and Mechanical University, Tallahassee, FL 32307, USA

^3^Water, Energy and Environmental Engineering Research Unit, Faculty of Technology, University of Oulu, 90014 Oulu, Finland

^4^Environmental Assessment Division, Indira Gandhi Centre for Atomic Research, Homi Bhaba National Institute, Kalpakkam, Tamil Nadu, India

^5^TNAU: Agricultural College & Research Institute - Madurai Campus, 625 104, India

^6^Water Management (Agriculture) Division, Centre for Water Resources Development and Management, Kunnamangalam-673571, Kozhikode, Kerala

^7^ICAR-Indian Institute of Soil and Water Conservation, R.C, Udhagamandalam-643 004, Tamil Nadu, India

**^*^Corresponding Author**

Dr. V Kumar, Former Professor and Head, Department of Agricultural Engineering, Madurai-625104, India Tel: 0091-452-4367104; Email: [vskumaran1955@gmail.com](mailto:vskumaran1955@gmail.com)

Section 1. Input Data to the DSSAT Crop Model

Table S1. The soil information at the Ludhiana (30°56′N, 75°52′E) station considered for the Punjab state in this study.

| **Depth (cm)** | **Bulk density (g cm^−3^)** | **FC (cm^3^ cm^−3^)** | **WP (cm^3^ cm^−3^)** | **Soil texture** | | | **pH (1:2)** |
| --- | --- | --- | --- | --- | --- | --- | --- |
|  |  |  |  | **Sand (%)** | **Silt (%)** | **Clay (%)** |  |
| 0–15 | 1.5 | 0.32 | 0.1 | 46 | 21.3 | 32.7 | 8.1 |
| 15–30 | 1.61 | 0.32 | 0.11 | 24 | 34.7 | 41.2 | 8.3 |
| 30–60 | 1.46 | 0.33 | 0.11 | 15.4 | 39.4 | 45.1 | 7.9 |
| 60–90 | 1.48 | 0.31 | 0.09 | 40 | 21.5 | 38.6 | 7.8 |
| 90–120 | 1.33 | 0.24 | 0.07 | 66.6 | 17.1 | 15.8 | 7.8 |
| 120–150 | 1.39 | 0.2 | 0.05 | 89.5 | 6.2 | 4.3 | – |
| 150–180 | 1.42 | 0.2 | 0.05 | 89.5 | 6.5 | 4.2 | – |

Table S2. The soil information for the Haryana state studied.

| **Station** | **Latitude (°N)** | **Longitude (°E)** | **Taxonomy** | **Depth** | **Soil Horizon** | **pH** | **EC** | **OC** | **CaCO3** | **Clay** | **Silt** | **Sand** | **CEC** | **BS** |
| --- | --- | --- | --- | --- | --- | --- | --- | --- | --- | --- | --- | --- | --- | --- |
| Kalka | 30.08 | 76.95 | Typic  Ustorthents | 0-13 | Ap | 7.3 | 0.06 | 0.49 | nil | 3.5 | 28.4 | 68.2 | 7.2 | 83 |
|  |  |  |  | 13-38 | A12 | 7.2 | 0.04 | 0.48 | nil | 7 | 24.4 | 68.6 | 8.6 | 80 |
|  |  |  |  | 38-70 | C1 | 7.3 | 0.02 | 0.4 | nil | 7 | 16.4 | 76.6 | 9.5 | 82 |
| Piruwala | 30.45 | 77.28 | Udic  Ustochrepts | 0-17 | Ap | 6.9 | 0.06 | 0.37 | nil | 4 | 36.4 | 49.6 | 6.36 | 94 |
|  |  |  |  | 17-50 | Bw1 | 7.1 | 0.02 | 0.26 | nil | 13.5 | 41.3 | 45.2 | 11 | 85 |
|  |  |  |  | 50-74 | Bw2 | 7 | 0.05 | 0.2 | nil | 14.5 | 39.1 | 46.4 | 13.6 | 88 |
|  |  |  |  | 74-136 | Bw3 | 7.5 | 0.02 | 0.18 | nil | 20.5 | 35.9 | 43.6 | 13.33 | 91 |
|  |  |  |  | 136-156 | Bw4 | 7.2 | 0.05 | 0.15 | nil | 28.5 | 37.9 | 23.6 | 15.91 | 87 |
| Bachhon | 30.66 | 77.45 | Typic  Ustifluvents | 0-16 | Ap | 7.8 | 0.12 | 0.58 | nil | 17 | 43.5 | 39.5 | 10.22 | 79 |
|  |  |  |  | 16-42 | C1 | 7.8 | 0.05 | 0.11 | nil | 7 | 12.2 | 80.8 | 5.25 | 78 |
|  |  |  |  | 42-66 | C2 | 7.7 | 0.05 | 0.09 | nil | 10 | 19.3 | 70.7 | 7.95 | 83 |
|  |  |  |  | 66-82 | C3 | 7.7 | 0.04 | 0.24 | nil | 16 | 43.6 | 40.4 | 11.01 | 81 |
|  |  |  |  | 82-103 | C4 | 7.4 | 0.04 | 0.26 | nil | 17 | 53.3 | 29.7 | 15.12 | 87 |
|  |  |  |  | 103-145 | C5 | 7.3 | 0.08 | nil | nil | 26 | 61.7 | 12.3 | 22.92 | 83 |
| Charkhidadri | 28.53 | 76.2 | Typic  Ustipsanments | 0-15 | Ap | 8.3 | 0.08 | 0.12 | nil | 4 | 9 | 87 | 3.26 | 63 |
|  |  |  |  | 15-65 | C1 | 8.8 | 0.12 | 0.1 | nil | 4 | 8.6 | 87.4 | 3.91 | 64 |
|  |  |  |  | 65-105 | C2 | 8.9 | 0.06 | 0.09 | nil | 5 | 9.6 | 85.4 | 4.77 | 68 |
|  |  |  |  | 105-150 | C3 | 9.3 | 0.15 | 0.08 | nil | 7 | 6.6 | 86.4 | 6.08 | 66 |
| Kot |  |  |  | 0-15 | Ap | 7.4 | 0.26 | 0.24 | nil | 27 | 21.5 | 51.5 | 9.62 | 84 |
|  |  |  |  | 15-39 | Bw1 | 7.3 | 0.76 | 0.18 | nil | 30 | 22.2 | 47.8 | 9.99 | 86 |
|  |  |  |  | 39-58 | Bw2 | 7.3 | 1.1 | 0.15 | nil | 28 | 24.6 | 47.4 | 9.92 | 88 |
|  |  |  |  | 58-82 | Bw3 | 7.6 | 1.3 | 0.11 | nil | 25 | 27.5 | 47.5 | 8.36 | 89 |
|  |  |  |  | 82-117 | C1 | 7.8 | 0.97 | 0.08 | nil | 19 | 22.8 | 52.5 | 9.03 | 49 |
|  |  |  |  | 117-160 | C2 | 8 | 1.1 | 0.06 | nil | 20 | 26.6 | 53.4 | 5.33 | 90 |

| Nangal |  |  |  | 0-19 | Ap | 8.5 | 0.11 | 0.18 | nil | 5.5 | 5 | 89.5 | 10.24 | 72 |
| --- | --- | --- | --- | --- | --- | --- | --- | --- | --- | --- | --- | --- | --- | --- |
|  |  |  |  | 19-35 | A12 | 8.7 | 0.1 | 0.9 | nil | 7 | 4.8 | 88.2 | 5.37 | 96 |
|  |  |  |  | 35-56 | C1 | 9.2 | 0.1 | 0.77 | nil | 4 | 7.3 | 88.7 | 5.37 | 99 |
|  |  |  |  | 56-90 | C2 | 9.5 | 0.1 | 0.03 | nil | 4 | 6 | 90 | 5.37 | 95 |
|  |  |  |  | 90-128 | C3 | 9.5 | 0.09 | 0.03 | nil | 5 | 4 | 91 | 4.7 | 100 |
| Mamoria |  |  |  | 0-16 | Ap | 7.6 | 0.12 | 0.24 | 1.43 | 12.5 | 11.76 | 75.74 | 7.94 | 87 |
|  |  |  |  | 16-30 | A11 | 7.7 | 0.21 | 0.24 | 0.11 | 11 | 6.9 | 82.1 | 6.4 | 86 |
|  |  |  |  | 30-60 | A12 | 7.8 | 0.13 | 0.24 | 0.22 | 16 | 7.08 | 76.92 | 5.89 | 73 |
|  |  |  |  | 60-91 | Bw1 | 8.3 | 0.05 | 0.11 | 0.44 | 14 | 7.52 | 78.48 | 6.66 | 88 |
|  |  |  |  | 91-124 | Bw2 | 8.5 | 0.05 | 0.19 | 0.55 | 16 | 7.27 | 76.73 | 7.2 | 97 |
|  |  |  |  | 124-160 | Bw3 | 8.7 | 0.05 | 0.11 | nil | 22.5 | 10.45 | 67.05 | 8.58 | 91 |
| Kulawar |  |  |  | 0-17 | Ap | 6.9 | 0.05 | 0.18 | nil | 6.5 | 36.5 | 47 | 6.82 | 82 |
|  |  |  |  | 17-60 | Bw1 | 7.9 | 0.04 | 0.2 | nil | 22.5 | 48.3 | 29.2 | 15.05 | 90 |
|  |  |  |  | 60-107 | Bw2 | 8 | 0.12 | 0.18 | nil | 25 | 52.3 | 22.6 | 17.25 | 89 |
|  |  |  |  | 107-142 | Bw3 | 8.1 | 0.24 | 0.14 | nil | 25 | 33.3 | 41.7 | 13.28 | 89 |
|  |  |  |  | 142-156 | Bw4 | 7.9 | 0.4 | 0.08 | nil | 13 | 28.1 | 58.9 | 10.37 | 100 |

Table S3. Physical and chemical properties for the different soils used in model evaluations and applications.

| **Depth (cm)** | **LL (cm^3^ cm^−3^)** | **DUL (cm^3^ cm^−3^)** | **SAT (cm^3^ cm^−3^)** | **SRGF** | **BD (Mg m^−3^)** | **SOC (%)** | **Clay (%)** | **Silt (%)** | **Sand (%)** |
| --- | --- | --- | --- | --- | --- | --- | --- | --- | --- |
| Experiments 1 and 2: sandy loam (PAU) | | | | | | | | | |
| 0–15 | 0.07 | 0.26 | 0.36 | 1 | 1.61 | 0.29 | 17.2 | 17.2 | 65.6 |
| 15–30 | 0.07 | 0.27 | 0.31 | 0.8 | 1.76 | 0.24 | 15.3 | 17.4 | 67.3 |
| 30–60 | 0.06 | 0.23 | 0.36 | 0.15 | 1.61 | 0.22 | 16.6 | 12 | 71.4 |
| 60–90 | 0.06 | 0.21 | 0.39 | 0.07 | 1.53 | 0.2 | 14.8 | 13 | 72.2 |
| 90–120 | 0.07 | 0.21 | 0.39 | 0.02 | 1.53 | 0.18 | 14 | 12.2 | 73.8 |
| 120–150 | 0.05 | 0.21 | 0.39 | 0.01 | 1.52 | – | 8.2 | 10.9 | 80.9 |
| 150–180 | 0.05 | 0.2 | 0.39 | 0.01 | 1.52 | – | 8.6 | 5.3 | 88.1 |
| Experiment 1: loam (Phillaur) | | | | | | | | | |
| 0–15 | 0.09 | 0.31 | 0.38 | 1 | 1.55 | 0.37 | 17.4 | 42.6 | 40 |
| 15–30 | 0.09 | 0.32 | 0.36 | 0.5 | 1.79 | 0.23 | 23.2 | 48.7 | 28.1 |
| 30–60 | 0.1 | 0.33 | 0.36 | 0.05 | 1.7 | 0.22 | 25 | 44 | 31 |
| 60–90 | 0.13 | 0.33 | 0.36 | 0.03 | 1.71 | 0.19 | 26 | 48.2 | 25.8 |
| 90–120 | 0.14 | 0.33 | 0.36 | 0.02 | 1.67 | 0.24 | 29.9 | 47.5 | 22.6 |
| 120–150 | 0.14 | 0.33 | 0.36 | 0.01 | 1.67 | – | 30.8 | 48.4 | 20.8 |
| 150–180 | 0.14 | 0.33 | 0.36 | 0 | 1.67 | – | 29.3 | 49.5 | 21.2 |
| Experiment 3: loamy sand (PAU) | | | | | | | | | |
| 0–8 | 0.07 | 0.26 | 0.44 | 1 | 1.6 | 0.42 | 24 | 10 | 66 |
| 9–17 | 0.07 | 0.25 | 0.38 | 0.8 | 1.64 | 0.4 | 25 | 11 | 64 |
| 18–22 | 0.06 | 0.25 | 0.35 | 0.15 | 1.62 | 0.4 | 25 | 12 | 63 |
| 23–37 | 0.05 | 0.25 | 0.36 | 0.07 | 1.63 | 0.22 | 24 | 19 | 57 |
| 38–55 | 0.06 | 0.24 | 0.36 | 0.02 | 1.62 | 0.32 | 23 | 15 | 62 |
| 56–72 | 0.06 | 0.25 | 0.39 | 0.01 | 1.56 | 0.2 | 25 | 16 | 59 |
| 73–89 | 0.06 | 0.24 | 0.38 | 0.01 | 1.54 | 0.11 | 25 | 16 | 59 |

Table S4. Physical and chemical characteristics of soil profiles at different locations in central Punjab.

| **Depth (cm)** | | **Sand (%)** | **Silt (%)** | **Clay (%)** | **CEC (meq)** | | **pH** | **Organic Matter (kg ha^−1^)** | **NO3**  **(kg ha^−1^)** | | **NH4**  **(kg ha^−1^)** |
| --- | --- | --- | --- | --- | --- | --- | --- | --- | --- | --- | --- |
| Amritsar (31°31′56″ N, 74°52′25″E) silt loam | | | | | | | | | | | |
| 0–15 | | 24.6 | 61.4 | 14 | 13.85 | | 8.8 | 0.91 | 25.2 | | 11.4 |
| 15–37 | | 21.5 | 57.9 | 21.8 | 14.21 | | 9.1 | 0.33 | 10.1 | | 8.3 |
| 37–50 | | 19.9 | 59.5 | 21.6 | 13.29 | | 9.2 | 0.19 | 5.9 | | 5.1 |
| 50–79 | | 18.7 | 59.9 | 21.5 | 13.33 | | 9.2 | 0.05 | 4.1 | | 3.3 |
| 79–106 | | 20.8 | 58.1 | 21.1 | 11.53 | | 9.2 | 0.09 | 3.3 | | 1.3 |
| 106–136 | | 12.8 | 70.2 | 17.2 | 13.34 | | 9.3 | 0.19 | 4.7 | | 3.6 |
| 136–162 | | 15.4 | 71.2 | 13.4 | 8.44 | | 9.3 | 0.12 | 6.7 | | 3.6 |
| 162–180 | | 15.4 | 71.2 | 13.4 | 8.44 | | 9.3 | 0.12 | 3.7 | | 2.5 |
| Jalandhar (31°31′50″N, 75°34′25″E) silt loam | | | | | | | | | | | |
| 0–12 | | 36.5 | 51.3 | 12.2 | 8.21 | | 8.96 | 0.88 | 19.8 | | 10.3 |
| 21–Dec | | 34.1 | 49.1 | 16.8 | 7.94 | | 9 | 0.47 | 11.3 | | 6.1 |
| 31–54 | | 27.5 | 51.5 | 21 | 11.27 | | 8.7 | 0.4 | 8.1 | | 4.4 |
| 54–75 | | 24.5 | 50.3 | 25.2 | 11.37 | | 8.62 | 0.38 | 5.3 | | 2.9 |
| 75–89 | | 22.5 | 48.7 | 28.8 | 13.26 | | 8.55 | 0.34 | 4.2 | | 2 |
| 89–127 | | 19.5 | 48.3 | 32.2 | 13.5 | | 8.6 | 0.31 | 6.1 | | 2.9 |
| 127–140 | | 35 | 44.8 | 20.2 | 9.37 | | 8.65 | 0.28 | 3.2 | | 2.4 |
| 140–180 | | 35 | 44.8 | 20.2 | 9.37 | | 8.65 | 0.28 | 3.2 | | 2 |
| Ludhiana (30°75′48″N, 75°48′30″E) sandy loam | | | | | | | | | | | |
| 0–21 | | 74.5 | 15.3 | 10.2 | 4.54 | | 8.3 | 0.6 | 12.1 | | 11.4 |
| 21–42 | | 53.3 | 34.9 | 11.8 | 7.09 | | 8.5 | 0.4 | 8.1 | | 5.2 |
| 42–65 | | 57.6 | 26.2 | 16.2 | 8.5 | | 8.4 | 0.34 | 9.4 | | 6.1 |
| 65–90 | | 57.3 | 25.1 | 17.5 | 8.6 | | 8.8 | 0.33 | 7.6 | | 4.3 |
| 90–116 | | 50.4 | 31 | 18.6 | 8.3 | | 8.2 | 0.31 | 4.8 | | 5.9 |
| 113–139 | | 50.9 | 30.5 | 18.6 | 9 | | 8.9 | 0.31 | 3.6 | | 4 |
| 139–180 | | 51 | 33.4 | 15.6 | 9.2 | | 8.9 | 0.29 | 2.8 | | 2.4 |
| Patiala (30°19′38″N, 76°24′00″E) loam | | | | | | | | | | | |
| 0–19 | 33.3 | | 48.7 | 18 | 7.99 | 8.6 | | 0.93 | 14.8 | 9.6 | |
| 19–53 | 21.8 | | 57.2 | 21 | 8.82 | 9.1 | | 0.14 | 7 | 5 | |
| 53–81 | 16.5 | | 59.1 | 24.4 | 9.3 | 9 | | 0.1 | 5.3 | 2.9 | |
| 81–102 | 17.6 | | 60 | 22.4 | 9.46 | 9 | | 0.16 | 5.3 | 2.9 | |
| 102–130 | 16.3 | | 58.3 | 25.4 | 10.8 | 8.9 | | 0.21 | 2.4 | 2.2 | |
| 130–180 | 16.3 | | 58.3 | 25.4 | 10.8 | 8.9 | | 0.21 | 1.9 | 1.9 | |

Table S5. Soil properties at the experimental sites in the Punjab and Haryana states studied.

| **Depth (cm)** | **Bulk density**  **(Mg m^−3^)** | **Clay (%)** | **Sand (%)** | **SWC at field capacity**  **(cm^3^ cm^−3^)** | **SWC at 15 bars**  **(cm^3^ cm^−3^)** | **pH (1:2)** | **Kunsatb**  **(1 kPa)**  **(mm h^−1^)** | **Kunsatb**  **(7 kPa)**  **(mm h^−1^)** | |
| --- | --- | --- | --- | --- | --- | --- | --- | --- | --- |
| Sandy loam | | | | | | | | | |
| 0–15 | 1.61 | 17.2 | 65.6 | 0.26 | 0.07 | 6.7 | 12 (15 cm) | 10 (15 cm) | |
| 15–30 | 1.76 | 15.3 | 67.3 | 0.27 | 0.07 | 7.3 | 25 (25 cm) | 14 (25 cm) | |
| 30–60 | 1.61 | 16.6 | 71.4 | 0.23 | 0.06 | 7.6 | 166 (45 cm) | 76 (45 cm) | |
| 60–90 | 1.53 | 14.8 | 72.2 | 0.21 | 0.06 | 7.7 |  | | |
| 90–120 | 1.53 | 14 | 73.8 | 0.21 | 0.07 | 7.8 | 183 (105 cm) | 171 (105 cm) | |
| 0–150 | 1.52 | 8.2 | 80.9 | 0.21 | 0.05 | NDc |  | | |
| 150–180 | 1.52 | 8.6 | 88.1 | 0.2 | 0.05 | NDc | 362 (165 cm) | 306 (165 cm) | |
| Silt loam | | | | | | | | | |
| 0–15 | 1.55 | 17.4 | 40 | 0.33 | 0.09 | 8.3 | 9 (15 cm) | 6 (15 cm) |  |
| 15–30 | 1.79 | 23.2 | 28.1 | 0.34 | 0.09 | 8.3 | 3 (25 cm) | 1 (25 cm) |  |
| 30–60 | 1.7 | 25 | 31 | 0.34 | 0.1 | 8.3 | 11 (45 cm) | 2 (45 cm) |  |
| 60–90 | 1.71 | 26 | 25.8 | 0.34 | 0.13 | 8.2 |  | | |
| 90–120 | 1.67 | 29.9 | 22.6 | 0.34 | 0.14 | 8.2 | 14 (105 cm) | 3 (105 cm) | |
| 120–150 | 1.67 | 30.8 | 20.8 | 0.34 | 0.14 | NDc |  | | |
| 150–180 | 1.67 | 29.3 | 21.2 | 0.34 | 0.14 | NDc | 8 (165 cm) | 2 (165 cm) | |

Table S6. Different management practices information for rice cultivation in both Punjab and Haryana states studied.

| **Planting time** | First fortnight of June |
| --- | --- |
| **Population** | 33 plants /hill |
| **Seeding depth** | 5 cm |
| **Row spacing** | 15-20 cm |
| **Irrigation dates** | Day 1(day of Transplantation) - 50 mm  20^th^ & 35^th^ day - 50mm  50^th^ & 60^th^ day - 80mm  85^th^ day - 60mm  100^th^ & 120^th^ day - 80mm |
| **Irrigation Type** | Surface irrigation Flooding |
| **Fertilizer** | Urea 120 kg /3 splits |
| **Time** | June 6^th^ – Sowing  June 26^th^ – Transplantation  November 1^st^ week – Harvest |

Table S7. The cultivar file (PR114) for rice in both Punjab and Haryana states studied.

| **Variable** | **Description** | **Coefficient Value** |
| --- | --- | --- |
| **P1** | Time Period (expressed as growing degree days in ⁰C above a base temperature of 9 ⁰C) from seedling emergence during which the rice plant is not responsive to changes in photoperiod. This period is also refered to as the basic vegetative phase of the plant. | 650 |
| **P2O** | Critical phoptoperiod or the longest day length (in hours) at which the development occurs at a maximum rate. At values higher than P2O, developmental rate is slowed, hence there is delay due to longer day lengths. | 200 |
| **P2R** | Extent to which phasic development leading to panicle initiation is delayed (expressed as growing degree days in ⁰C) for each hour increase in photoperiod above P2O | 520 |
| **P5** | Time period in growing degree days in ⁰C from beginning of grain filling (3 to 4 days after flowering) to physiological maturity with a base temperature of 9 ⁰C. | 12 |
| **G1** | Potential spikelet number coefficient as estimated from the number of spikelets per g of main culm dry weight (less lead blades and sheaths plus spikes) at anthesis. A typical value is 55. | 59 |
| **G2** | Single grain weight (g) under ideal growing conditions, i.e. nonlimiting light, water, nutrients, and absence of pests and diseases. | 0.025 |
| **G3** | Tillering coefficient (scalar value) relative to IR64 cultivar under ideal conditions. A higher tillering cultivar would have a coefficient greater than 1.0 | 1.0 |
| **G4** | Temperature tolerance coefficient. Usually 1.0 for varieties grown in normal environments. G4 for japonica type rice growing in a warmer environment would be 1.0 or greater. Likewise, the G4 value for indicak type rice in very cool environments or season would be less than 1.0 | 1.0 |

**Source:** Pathak, H., Timsina, J., Humphreys, E., Godwin, D.C., Bijay-Singh, Shukla, A.K., Singh, U., Matthews, R.B., 2004. Simulation of rice crop performance and water and N dynamics, and methane emissions for rice in northwest India using CERES-Rice model. In: CSIRO Land and Water Technical Report 23/04. CSIRO Land and Water, Griffith, NSW 2680, Australia, pp. 118.


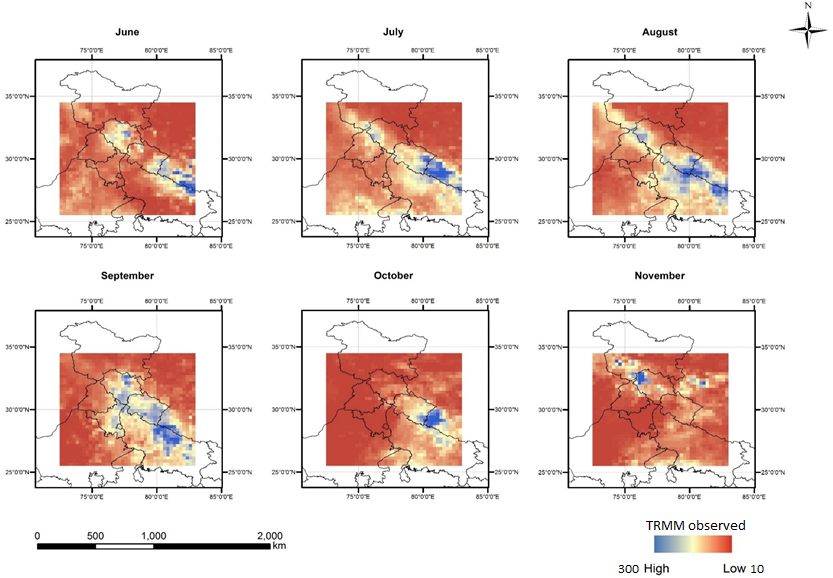


Figure S1. Spatio-temporal pattern of TRMM observed monthly rainfall (mm) during the months Jun-Nov in 2009 over both Punjab and Haryana states. Map created at IIRS - https://www.iirs.gov.in/inhouselaboratories


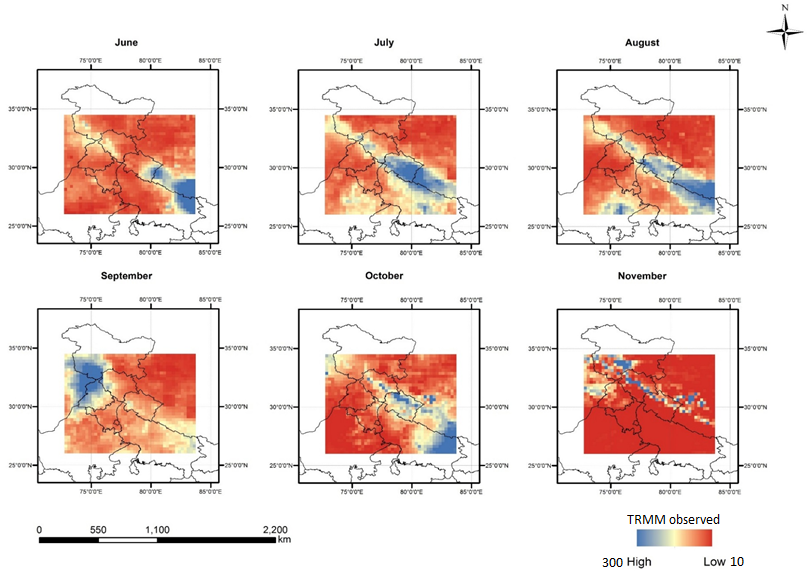


Figure S2. Spatio-temporal pattern of TRMM observed monthly rainfall (mm) during the months Jun-Nov in 2014 over both Punjab and Haryana states. (Map created at IIRS - https://www.iirs.gov.in/inhouselaboratories)


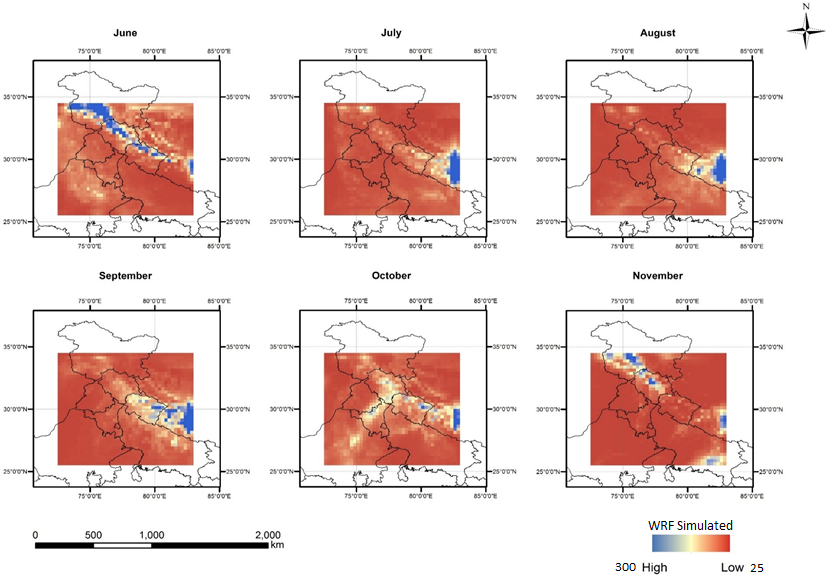


Figure S3. Spatio-temporal pattern of WRF simulated monthly rainfall (mm) during the months Jun-Nov in 2009 over both Punjab and Haryana states. Map created at IIRS - https://www.iirs.gov.in/inhouselaboratories


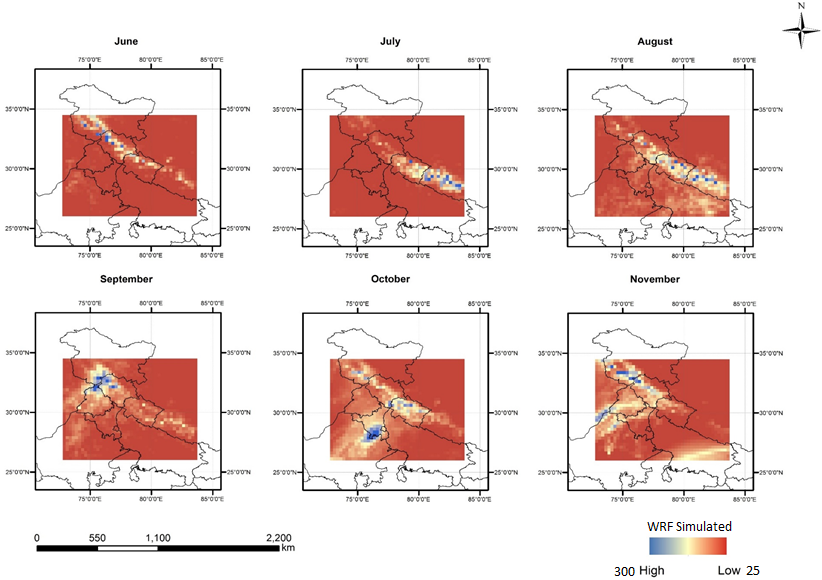


Figure S4. Spatio-temporal pattern of WRF simulated monthly rainfall (mm) during the months Jun-Nov in 2014 over both Punjab and Haryana states. Map created at IIRS - https://www.iirs.gov.in/inhouselaboratories

**
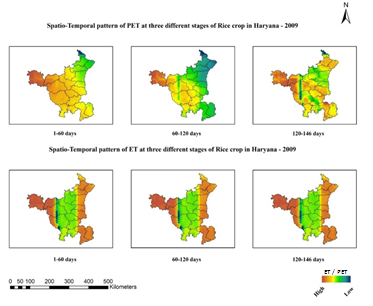

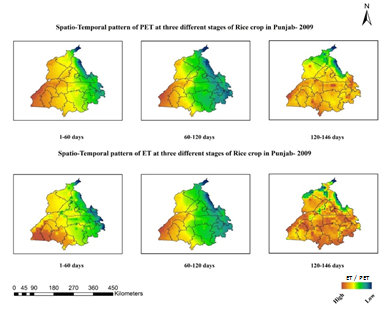
**

Figure S5. Spatio-temporal pattern map of (a) PET and (b) ET, during the three stages of rice crop growth across Haryana and Punjab in 2009. Map created at IIRS - https://www.iirs.gov.in/inhouselaboratories.


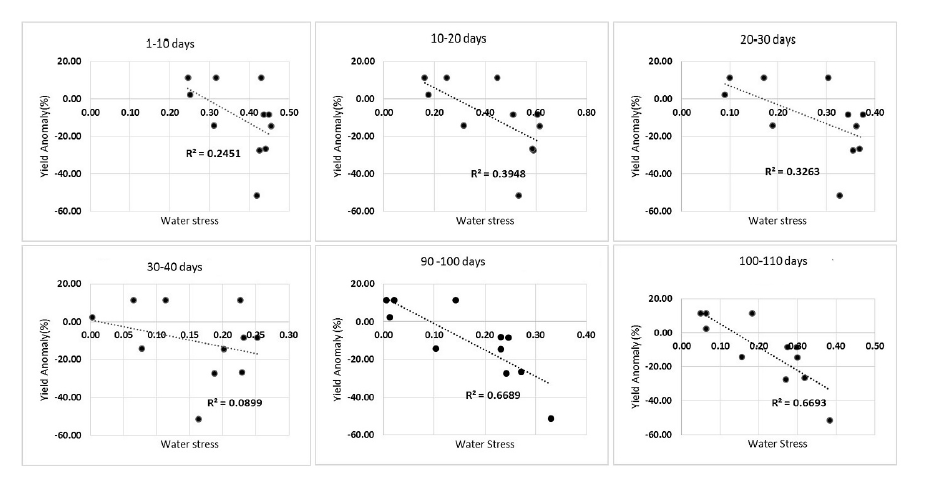
Figure S6. The impacts of rice crop water stress factor on annual rice yield in Haryana during the vegetative (a) 1-10 days, (b) 10-20 days, (c) 20-30 days, and (d) 30-40 days and reproductive (e) 90-100 days and (f) 100-110 days stages in 2009.
